# Temporal Properties of Cardiorespiratory Coupling in Patients with Heart Failure During the Circadian Cycle

**DOI:** 10.3390/e28050524

**Published:** 2026-05-06

**Authors:** Natalia Buitrago-Ricaurte, José Javier Reyes-Lagos, Karsten Berg, Rafael González Niño, Thomas Penzel, Niels Wessel

**Affiliations:** 1 Escuela de Medicina y Ciencias de la Salud, Universidad del Rosario, Bogotá 11221, Colombia; natalia.buitragor@urosario.edu.co; 2 Sección de Bioelectrónica, Departamento de Ingeniería Eléctrica, Centro de Investigación y de Estudios Avanzados del Instituto Politécnico Nacional, Gustavo A. Madero, Ciudad de México, Mexico City 07360, Mexico; javier.reyes@cinvestav.mx; 3 Department of Physics, Humboldt-Universität zu Berlin, 10099 Berlin, Germany; khgberg@physik.hu-berlin.de; 4 Cardiology Department, Instituto del Corazón de Bucaramanga-Sede Bogotá, Bogotá 111411, Colombia; rafaelgonzaleznino@gmail.com; 5 Center for Sleep Medicine, Charité–Universitätsmedizin Berlin, 10117 Berlin, Germany; thomas.penzel@charite.de; 6 Department of Human Medicine, MSB Medical School Berlin GmbH, 14197 Berlin, Germany

**Keywords:** heart failure, circadian rhythm, autonomic regulation, cardiorespiratory coupling, entropy, nonlinear dynamics

## Abstract

Heart failure (HF) is accompanied by autonomic dysregulation and disrupted physiological rhythms, yet how cardiorespiratory coupling (CRC) reorganizes across the circadian cycle during everyday life remains incompletely characterized. We studied 24 h ambulatory ECG recordings from 88 healthy controls and 75 patients with HF. Cardiac autonomic dynamics were quantified from RR intervals using standard HRV indices and symbolic/entropy descriptors, and circadian organization was assessed with 24 h cosinor modeling. Respiratory timing was derived from ECG-derived respiration (EDR) to obtain breath-to-breath (BB) intervals and the pulse–respiration quotient (PRQ). System-level coupling was evaluated primarily as event-timing coordination using coordigram-based coordination percentages computed at two timing tolerances (ε = 0.1 s and ε = 0.2 s) over 24 h and hour-by-hour, and complemented by entropy-based timing irregularity for RR, BB, PRQ, and RR–BB cross-entropy. Patients with HF exhibited lower global HRV and reduced information content in RR dynamics, together with circadian chronodisruption characterized mainly by weaker rhythmic expression and increased inter-individual phase dispersion. CRC differences depended on both tolerance and time of day. In entropy-based profiles, RR–BB cross-entropy and RR entropy did not show hour-specific differences; instead, group separation was localized to higher early-night ApEn in BB in HF and to consistently higher daytime/early-evening FuzzEn in PRQ in controls. Together, these findings indicate a time-structured remodeling of cardiac autonomic dynamics and CRC in HF, in which autonomic function is compromised, and coupling alterations become most evident when examined at appropriate timing tolerances and with circadian (hour-resolved) resolution.

## 1. Introduction

Cardiovascular and respiratory function exhibits oscillatory activity across the 24 h cycle, which confers resilience to short-term challenges while maintaining operation within a stable physiological threshold [1]. In parallel, respiratory function also follows circadian oscillations, with time-of-day-dependent modulation of airway mechanics, ventilatory control, and pulmonary immune activity, which shapes adaptive respiratory responses across the 24 h cycle [2]. In the short term, the heart exhibits rhythmic electrical activity that allows for flexible, dynamic conduction–contraction coupling in response to metabolic, hemodynamic, and autonomic challenges. These temporal dynamics across short- and long-term windows enable the system to synchronize with internal and external cues to optimize resource allocation, maintain homeostasis, and adapt to environmental demands [3]. The study of temporal properties requires evaluating physiological descriptors such as complexity, repertoire, and divergence, which reflect how flexible, rich, and robust the system’s responses are over time [4,5]. Interpreting cardiac electrical and respiratory activity through the autonomic function embedded in their temporal patterns is essential for understanding their physiological implications in both health and disease [6,7].

In pathological states, the temporal structure of cardiac and respiratory rhythms, often indexed by heart and respiratory rate variability, becomes dysregulated, reflecting maladaptive responses to metabolic, hemodynamic, and neurohumoral stressors and is associated with impaired oxygenation and clinical deterioration [8,9,10]. The electrical heart signals in potentially modifiable conditions such as diabetes, coronary artery disease, chronic respiratory disease, and heart failure exhibit reduced complexity and repertoire, as well as attenuated circadian modulation [11,12,13]. These alterations could be explained by reduced autonomic flexibility, impaired entrainment mechanisms, and disrupted neurohumoral regulation [11,12]. Aging as a key non-modifiable factor, also exerts a marked effect on the temporal properties of cardiac and respiratory signals; with advancing age, these temporal dynamics evolve toward blunted amplitudes, reduced variability, and loss of rhythmic complexity [13,14].

Heart failure (HF) is a highly prevalent disease with impairments in the heart’s contractility, electrical properties, and autonomic regulation driven by modifiable and non-modifiable factors that, over a chronic timeline, give rise to maladaptive processes. Activation of the renin–angiotensin system, the shift toward anaerobic metabolism, and autonomic imbalance lead to remodeled cardiac tissue with altered contractile and electrical properties, resulting in pump dysfunction, arrhythmogenesis, and impaired hemodynamic regulation [15]. Particularly, arrhythmogenesis increases mortality and adverse outcomes in patients with HF. It is well known that remodeled hearts exhibit altered conduction pathways, heterogeneous repolarization, and increased fibrosis, which lead to electrical instability and reentrant circuits, ultimately resulting in atrial fibrillation and a range of ventricular arrhythmias [12,16].

Beyond contractile and electrical remodeling, HF is also accompanied by altered interactions between cardiac and respiratory rhythms [17]. Cardiorespiratory coupling (CRC) describes how cardiac and respiratory rhythms interact across behavioral states and mechanisms, providing an integrative window into regulation of autonomic networks and their changes across the health–disease continuum [18]. One common mode of CRC is event-timing coordination, i.e., the temporal alignment of cardiac events with respiratory events. In this study, we assess CRC primarily through coordigram-based event-timing coordination, and we complement this view with entropy-based descriptors of cardiac, respiratory, and integrative timing dynamics. For example, in HF with reduced ejection fraction, CRC indices have been linked to myocardial recovery phenotypes [19], and nonlinear symbolic dynamics applied to cardiac and respiratory interval series can distinguish abnormal breathing patterns in chronic HF, underscoring the potential value of interaction-aware metrics [20]. However, existing CRC studies on HF are limited by small cohorts, heterogeneous signal-processing methods, and a focus on cross-sectional associations rather than mechanistic analysis.

Cardiac arrhythmias affect CRC, altering the temporal structure of respiratory activity and potentially contributing to the unstable coupling observed in HF [21]. Cardiac arrhythmias are temporal alterations that reflect the functional, contractile, and autonomic disturbances characteristic of HF. It is known that arrhythmias increase heart rate variability (HRV), signal complexity, and the unpredictability of cardiac electrical activity. Unfortunately, arrhythmias may be asymptomatic, not sustained, and challenging to detect in many cases. Secondly, the temporal dynamics of cardiac activity across the circadian cycle in patients with heart failure remain poorly characterized [12]. Thirdly, there is a limited understanding of how these temporal disturbances relate to underlying autonomic dysregulation, cardiorespiratory coupling, and disease progression [22].

Therefore, we pose the following research question: What are the temporal properties of circadian cardiorespiratory coupling in heart failure? Our motivation is to identify critical time-of-day windows of increased vulnerability that could aid early diagnosis and refine therapeutic strategies. Accordingly, we aimed to (i) describe 24 h patterns in cardiac autonomic dynamics using entropy and symbolic descriptors; (ii) characterize circadian alterations in these temporal metrics; and (iii) assess whether changes in cardiac temporal organization are accompanied by disrupted CRC and increased irregularity of cardiorespiratory rhythms. This work has important implications for advancing the characterization of autonomic chronodisruption in HF by integrating CRC and rhythm irregularity into a 24 h framework, thereby providing temporal biomarkers for clinical risk stratification and supporting the development of chronobiology-informed therapeutic interventions.

## 2. Materials and Methods

### 2.1. Subjects

We conducted an observational retrospective study analyzing data from 163 individuals (88 controls and 75 patients with heart failure). Controls were selected from the Telemetric and Holter ECG Warehouse (THEW) dataset, which contains continuous electrocardiographic recordings and clinical information from multiple studies, including healthy volunteers [23]. We performed an a priori power analysis using G*Power 3 (Version 3.1.9.7 (Heinrich Heine University Düsseldorf, Düsseldorf, Germany)), which indicated that a total sample size of 160 would achieve 0.95 statistical power at an alpha level of 0.05 to detect a medium effect size in an independent-samples *t*-test. For the power analysis method, we followed the method of Faul et al. [24]. HF group participants were outpatients at the Instituto del Corazón de Bucaramanga, Sede Bogotá. Groups were selected based on the reported inclusion criteria [25].

Patients with HF were excluded if the following conditions were reported in clinical records or identified in the 24 h ECG recording: atrial fibrillation, presence of a pacemaker or implantable cardioverter-defibrillator, significant arrhythmias (frequent premature atrial or ventricular contractions, supraventricular or ventricular tachycardia), moderate-to-severe valvular heart disease, chronic pulmonary disease (COPD), interstitial lung disease, and diagnosed sleep apnea. Additional exclusions included poor ECG signal quality and incomplete recordings.

Recordings from the individuals in the control group were obtained using the SpaceLab-Burdick digital Holter recorder (SpaceLab-Burdick, Inc., Deerfield, WI, USA), while recordings from the patients with heart failure were acquired using the Sentinel-Pathfinder SL^®^ (Spacelabs Healthcare, Snoqualmie, WA, USA, V.9800). Both devices operated at a sampling rate of 150 Hz and 12-bit ADC. A direct sensitivity analysis of higher-sampling-rate recordings was not feasible because the acquisition system operates at a fixed sampling rate of 150 Hz. Therefore, higher-resolution versions of the same signals were not available for reanalysis. This should be considered when interpreting the results.

Recordings started with a 20 min supine baseline, followed by a continuous 24 h period during free-routine activity. This study received approval from the Ethical Committee of the Instituto del Corazón de Bucaramanga, Sede Bogotá, Colombia (protocol number 06, 8 November 2023). Electrocardiogram activity was monitored over 24 h in ambulatory conditions using three leads with a pseudoorthogonal configuration (X, Y, and Z) [26]. The sampling frequency was 150 Hz, and the amplitude resolution was 10μV. The recordings were visually supervised and manually de-artifacted by a specialized technician using Vision Premier (Vision Premier version 3.5, SpaceLab-Burdick, Inc., Deerfield, WI, USA).

### 2.2. Preprocessing of ECG Signals and RR Extraction

**Figure 1:**
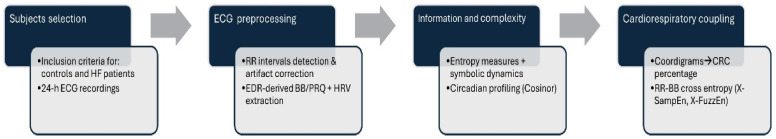
Overview of the analysis pipeline from 24 h ECG recordings. After subject selection, ECG signals were quality-controlled to extract RR intervals and EDR-derived BB/PRQ series. Autonomic and complexity metrics were computed, and their 24 h rhythmicity was assessed using cosinor analysis. Cardiorespiratory coupling was quantified using coordigrams and RR–BB cross-entropy (X-SampEn and X-FuzzEn). Primary outcomes: CRC% ε = 0.2 h; FuzzEnPRQ hourly; ApEnBB hourly; acrophase. Secondary/supportive outcomes: HRV; symbolic and entropy metrics.

ECG preprocessing followed the established guidelines [27,28]. We assessed RR-interval signals by identifying and removing artifacts using noise detection and threshold-based beat correction in Kubios (v. 4.3.1; Kubios, OY, Kuopio, Finland). Of the 402 eligible recordings, we excluded nearly 60% due to incomplete recordings, strong threshold beat correction (defined as >5% of total beats), and an ectopy rate >10% (Figure S1). Five analyzed recordings required beat correction with corrected heartbeats within 1–3%. We agree that artifact and ectopic beat removal alone does not ensure stationarity. To address this, we segmented data into short windows (5 min) and applied Kubios detrending (smoothness priors). Thus, approximate local stationarity was assumed, consistent with HRV standards. Finally, we selected free-artifact windows for 5 min per hour based on the first clean segment for time-domain and frequency-domain parameters, and 30 min per hour for symbolic dynamics metrics (Figure 1). To enhance data quality, only segments with minimal correction (≤3% corrected beats in included recordings) were retained for analysis.

### 2.3. ECG-Derived Respiration (EDR), Breath-to-Breath Intervals (BB), and PRQ Computation

ECG-derived respiration (EDR) was obtained from beat-to-beat modulation of the R-wave amplitude, following published amplitude-based approaches to ECG-derived respiration [29,30]. In brief, the ECG was filtered to remove power-line interference and reduce baseline wander, and R peaks were detected using an automated algorithm [31]. R-peak amplitudes were sampled at the detected R-peak locations to obtain support points for EDR. After outlier removal, these support points were interpolated using piecewise cubic Hermite interpolation to reconstruct a continuous surrogate respiratory waveform. The reconstructed EDR signal was then band-pass-filtered (0.05–0.8 Hz is the default setting of the reconstruction routine) and used to identify respiratory onsets and derive the breath-to-breath (BB) interval series. Previous work has shown that amplitude-based EDR can provide a clinically useful surrogate of respiratory dynamics in several settings, including sleep-related and heart-failure-associated breathing assessment. However, because no simultaneously recorded reference respiratory signal was available in the present dataset, EDR should be interpreted as a practical surrogate respiratory waveform for timing analysis rather than a validated substitute for direct respiratory measurement [32].

Respiratory onsets were operationally defined as the time instants corresponding to the negative zero-crossings of the second derivative of the EDR signal. This onset definition was used as a reproducible timing rule for deriving BB intervals and subsequent coordination analysis, and should be understood as an implementation-specific choice within the present CRC framework. Alternative respiratory event definitions may yield slightly different BB timing markers.

Cardiac timing was represented by the RR interval series obtained from the same ECG recordings. The instantaneous pulse–respiration quotient (PRQ) series was computed as the ratio of cardiac to respiratory rates [33], derived from RR and BB, and was analyzed alongside the individual RR and BB series. Additionally, hourly respiratory rate (RPM) was derived from BB intervals (BR = 60/BB) and summarized per hour; results are reported in the Supplementary Materials (Figure S3).

### 2.4. Time and Frequency Domain

Time- and frequency-domain HRV parameters were analyzed following previously validated methodologies. A total of thirteen time-domain (SDNN, cvNN, SDANN1, SDANN5, SDANN10, RMSSD, pNNl10, pNNl20, pNNl30, pNNl50, pNN50 pNN100, and pNN200) and thirteen frequency-domain HRV indices (Total Power-TP, ULF, VLF, LF/HF, LF/TP, HF/TP, VLF/TP, ULF/TP, ULF + VLF + LF/TP, ULF + VLF/TP, UVLF, LFn, and HFn) were analyzed in accordance with standard recommendations and prior publications (Task Force) [28]. These parameters were selected for their potential to characterize global autonomic modulation, sympathetic–parasympathetic balance, and oscillatory components of cardiac autonomic regulation.

### 2.5. Symbolic Dynamics

We applied the previously published approach to identify symbolic dynamics [34]. RR interval series were transformed into symbolic sequences using an alphabet size of four symbols based on predefined thresholds of interval variation. Symbolic words were then constructed using a word length of three consecutive symbols, a configuration widely used to balance sensitivity to dynamic changes and robustness. From this symbolic representation, we quantified the distribution of word patterns using the following metrics:

- FORBWORD: The number of word types that occur with a probability of less than 0.001 reflects the uncertainty and information content of the symbolic sequence.
- WSDVAR: Variance in the symbolic word distribution, which reflects the heterogeneity of symbolic pattern occurrence.
- WPSUM02: Sum of probabilities of symbolic words belonging to low-variability pattern classes, associated with regular dynamics.
- WPSUM13: Sum of probabilities of symbolic words belonging to low-variability pattern classes, associated with more complex dynamics.
- PLVAR: Percentage of symbolic patterns classified within variability classes (5= intermediate; 10 = high and 20 = higher). PHVAR: percentage of symbolic patterns classified within variability classes (20 = high and 50 = very high).
- FWSHANNON: Shannon entropy of the symbolic word distribution quantifies the overall uncertainty and information content of the symbolic sequence.
- Rényi entropy: (FWRENYI α = 0.25; α = 2 and α = 4) gives the greater weight to frequent symbolic patterns.

### 2.6. Cardiorespiratory Coupling Assessed as Event-Timing Coordination

In this work, CRC was operationalized primarily as event-timing coordination between R-peaks and respiratory onsets, quantified as the percentage of coordinated beats within ±ε (ε = 0.1 s and 0.2 s).

Event-timing coordination was quantified using coordigrams, i.e., two-dimensional maps of the temporal relationship between cardiac and respiratory events [35,36]. First, we obtained the timestamps of all R peaks $t_{R}(k)$ and all respiratory onsets $t_{A}(j)$ from the EDR signal. Respiratory onsets were identified as the negative zero crossing of the second derivative of the respiratory waveform around its maxima, which provides robust timing of the transition from inspiration to expiration.

For each heartbeat, we computed the time difference Δt between the R peak and its nearest respiratory onset within a range spanning two respiratory cycles. Plotting Δt (y-axis) versus time or breath index (x-axis) produces a point representation of CRC. To obtain a smooth representation, we estimated, for each sliding window of five consecutive respiratory onsets, the distribution of Δt using kernel density estimation (bandwidth = 0.1 s; Δt axis from −8 to 8 s in steps of 0.05 s). Stacking these distributions over time yields the coordigram, which was used as a smoothed visualization of the temporal coordination structure between cardiac and respiratory events. The x-axis represents time (or breath number), the y-axis represents the lag Δt between cardiac and respiratory events, and the color encodes the probability density. The primary quantitative CRC indices reported in this study were derived from the epsilon-strip coordination curve and not directly from the kernel density estimate itself. Positive ￼Δt values correspond to heartbeats following respiratory onsets, whereas negative values correspond to heartbeats preceding respiration.

To summarize, CRC from the coordigram was obtained using an epsilon-strip approach inspired by Berg’s method [37]. For a set of tolerance values ε (in seconds), we computed the proportion of heartbeats with ∣Δt∣that remained within the horizontal band [−*ε*, +*ε*]. This proportion, expressed as a percentage of all beats, defines the coordination curve C(ε) that quantifies how closely the cardiac rhythm is coupled to respiration across different coordination tolerances. From $C\left(\varepsilon \right)$, we extracted summary indices at selected tolerances (e.g., ε=0.1 s and ε=0.2 s), representing the percentage of “coordinated” beats for narrow and wider coordination windows. These values were used as empirically selected representative timing windows within the epsilon-strip framework and were not intended as clinical cutoffs. Importantly, ε was used only for coordigram-based coordination analysis and did not enter the entropy-based complexity analysis, which relied on separate embedding and tolerance parameters, as described in Section 2.7. These indices were computed for each one-hour segment across the 24 h recording and also over the full 24 h time series and then used as scalar measures of CRC.

### 2.7. Entropy-Based Complexity Analysis of Cardiac, Respiratory, and Cardiorespiratory Timing

To analyze the nonlinear variability in cardiac, respiratory, and cardiorespiratory timings, we calculated approximate entropy (ApEn), sample entropy (SampEn), and fuzzy entropy (FuzzEn) for the RR, BB, and PRQ time series with the EntropyHub toolbox in MATLAB R2024a (The MathWorks Inc., Natick, MA, USA) [38]. These measures were used as complementary rather than interchangeable estimators. ApEn was retained as a classical reference measure of time-series regularity following Pincus [39]; SampEn was used as a less biased alternative that avoids self-matches following Richman and Moorman [4]; and FuzzEn was used as a smoother similarity-based estimator. This combined approach allowed us to determine whether group differences were consistent across estimators or specific to a particular entropy definition. To quantify the CRC, we additionally computed cross-sample entropy (X-SampEn) and cross-fuzzy entropy (X-FuzzEn) between the RR and BB series.

Entropy was calculated using an hourly, circadian-resolved workflow. Recordings were divided into consecutive 1 h segments, and entropy was measured within each segment to observe daily fluctuations in complexity. Before estimating entropy, the time series were detrended to reduce slow drifts and nonstationarity, then normalized to allow comparable tolerance thresholds across subjects and windows. Since entropy algorithms can be computationally intensive for long sequences, RR, BB, and PRQ were analyzed as uniformly sampled surrogate signals. The saved signals were available at 4 Hz, and when the BB and PRQ time vectors did not exactly match the RR time vector, they were aligned to the RR master time using piecewise cubic Hermite interpolation. Within each hourly window, the 4 Hz signals were then decimated by a factor of 4, yielding 1 Hz representations for entropy estimation. Accordingly, entropy measures were computed on uniformly sampled representations of the interval-derived signals rather than on the original unevenly spaced event series. This approach was chosen to provide synchronized time series of equal length for both single-series and cross-entropy analyses while keeping the computation tractable over 24 h hourly windows. Only segments that met a minimum coverage threshold of at least 90% of the expected samples after downsampling were retained. In the hourly workflow, this corresponded to an expected length of 3600 samples per window at 1 Hz and a minimum required length of 3240 valid samples for each series (RR, BB, and PRQ).

Let $x=\{x_{1},\ldots ,x_{N}\}$ denote a preprocessed physiological time series. Delay-embedded template vectors of length m and delay τ were formed as follows:

(1) $$x_{i}^{\left(m\right)}=\left[x_{i},\;x_{i+\tau },\;\ldots ,\;x_{i+\left(m-1\right)\tau }\right],\;i=1,\ldots ,N_{m}$$

Template similarity was quantified using the Chebyshev distance:

(2) $$d_{ij}^{\left(m\right)}=\underset{k=0,\ldots ,m-1}{\max}\left|x_{i+k\tau }-x_{j+k\tau }\right|$$

Across all entropy metrics, we used standard physiological parameter choices: m=2 and τ=1. These values represent conventional analysis settings applied consistently across subjects and groups to ensure comparability and reproducibility, and were not intended to reflect a parameter optimization procedure for RR dynamics in heart failure [4,40,41,42]. Here, *τ* denotes the embedding delay in samples of the uniformly sampled surrogate signals used for entropy estimation. In the hourly analysis, after decimation to 1 Hz, *τ* = 1, which corresponds to 1 s. The tolerance (*r*) was set to a fixed fraction of the within-window variability:

(3) $$r=0.2\;\mathrm{SD}\left(x\right)$$

Approximate entropy (ApEn) [39]

For ApEn, the fraction of templates $x_{j}^{\left(m\right)}$ within distance r of $x_{i}^{\left(m\right)}$ was computed as follows:

(4) $$C_{i}^{\left(m\right)}\left(r\right)=\frac{1}{N_{m}}\sum_{j=1}^{N_{m}}1\left(d_{ij}^{\left(m\right)}\leq r\right)$$

The average log-likelihood of pattern similarity was then calculated:

(5) $$\phi^{\left(m\right)}\left(r\right)=\frac{1}{N_{m}}\sum_{i=1}^{N_{m}}\ln\;\left(C_{i}^{\left(m\right)}\left(r\right)\right)$$

Finally, ApEn was defined as follows:

(6) $$\mathrm{ApEn}\left(m,\tau ,r\right)=\phi^{\left(m\right)}\left(r\right)-\phi^{\left(m+1\right)}\left(r\right)$$

Sample entropy (SampEn) [4]

To reduce finite-sample bias, SampEn excluded self-matches (j≠i). We computed the match probability for templates of length m:

(7) Bmr=1NmNm−1∑i=1Nm ∑j=1j≠iNm1dijm≤r

and for templates of length m+1:

(8) Amr=1Nm+1Nm+1−1∑i=1Nm+1 ∑j=1j≠iNm+11dijm+1≤r

SampEn was then calculated:

(9) $$\mathrm{SampEn}\left(m,\tau ,r\right)=-\ln\;\left(\frac{A^{\left(m\right)}\left(r\right)}{B^{\left(m\right)}\left(r\right)}\right)$$

Fuzzy entropy (FuzzEn) [43]

FuzzEn replaces the hard thresholding in Equations (4), (7) and (8) with a smooth fuzzy membership function. Using an exponential membership with exponent n (EntropyHub), we defined the following:

(10) $$\mu \left(d;r,n\right)=\exp\;\left(-{\left(\frac{d}{r}\right)}^{n}\right)$$

The average fuzzy similarity for templates of length m was as follows:

(11) Φmr,n=1NmNm−1∑i=1Nm ∑ j=1,j≠iNmμdijm;r,n

and FuzzEn was calculated as follows:

(12) $$\mathrm{FuzzEn}\left(m,\tau ,r,n\right)=\ln\;\left(\frac{\Phi^{\left(m\right)}\left(r,n\right)}{\Phi^{\left(m+1\right)}\left(r,n\right)}\right)$$

In practice, entropy values were obtained using EntropyHub’s implementations of ApEn, SampEn, and FuzzEn; for FuzzEn, we used the EntropyHub default fuzzy function with exponent n=2.

Cross-entropy

To characterize CRC beyond event-timing coordination, cross-entropy was computed between aligned RR and BB interval-derived surrogate signals represented on a common temporal grid by comparing embedded vectors across series. The cross-series distance was defined as follows:

(13) $$d_{ij,xy}^{\left(m\right)}=\underset{k=0,\ldots ,m-1}{\max}\left|x_{i+k\tau }-y_{j+k\tau }\right|$$

Accordingly, the indices in Equation (13) refer to synchronized sample positions in the aligned RR and BB surrogate signals, rather than to raw beat-to-beat and breath-to-breath event sequences.

For cross-entropy, tolerance was defined in our implementation from the pooled variability of the concatenated RR and BB samples within each window:

(14) $$r_{\mathrm{pool}}=0.2\;\mathrm{SD}\left(\left[x;y\right]\right)$$

In our workflow, RR and BB were first represented as uniformly sampled surrogate signals on a common temporal grid; in the final version used for entropy analysis, both series were mean-centered and detrended before cross-entropy estimation. Because RR and BB were expressed in the same units (seconds), this implementation did not involve a unit mismatch. However, this pooled-tolerance formulation is implementation-specific and should not be interpreted as a full variance normalization of each series.

Cross-sample entropy (X-SampEn) used the following cross-match probabilities [4]:

(15) $$B_{xy}^{\left(m\right)}\left(r\right)=\frac{1}{N_{x,m}N_{y,m}}\sum_{i=1}^{N_{x,m}}\sum_{j=1}^{N_{y,m}}1\left(d_{ij,xy}^{\left(m\right)}\leq r\right)$$

(16) $$A_{xy}^{\left(m\right)}\left(r\right)=\frac{1}{N_{x,m+1}N_{y,m+1}}\sum_{i=1}^{N_{x,m+1}}\sum_{j=1}^{N_{y,m+1}}b1!\left(d_{ij,xy}^{\left(m+1\right)}\leq r\right)$$

(17) $$X\text{-}\mathrm{SampEn}\left(m,\tau ,r\right)=-\ln\;\left(\frac{A_{xy}^{\left(m\right)}\left(r\right)}{B_{xy}^{\left(m\right)}\left(r\right)}\right)$$

Analogously, cross-fuzzy entropy (X-FuzzEn) used the following fuzzy similarities [44]:

(18) $$\Phi_{xy}^{\left(m\right)}\left(r,n\right)=\frac{1}{N_{x,m}N_{y,m}}\sum_{i=1}^{N_{x,m}}\sum_{j=1}^{N_{y,m}}\mu \left(d_{ij,xy}^{\left(m\right)};r,n\right)$$

(19) $$X\text{-}\mathrm{FuzzEn}\left(m,\tau ,r,n\right)=\ln\;\left(\frac{\Phi_{xy}^{\left(m\right)}\left(r,n\right)}{\Phi_{xy}^{\left(m+1\right)}\left(r,n\right)}\right)$$

All cross-entropy values were obtained using EntropyHub’s XSampEn and XFuzzEn functions, using the same m, τ, and the pooled-tolerance implementation described in Equation (14).

These entropy-based cross-series measures were used to characterize shared temporal regularity between RR and BB surrogate signals and should not be interpreted as directed or causal interaction metrics.

No additional post hoc finite-sample bias correction was applied beyond the standard definitions of the entropy estimators used here. In particular, SampEn was computed in its self-match-excluding form, whereas ApEn, FuzzEn, XSampEn, and XFuzzEn were obtained directly from their standard implementations.

### 2.8. Analysis

We defined the analysis groups as “control” and “heart failure”. We also included the following variables for further analysis: age, sex, body mass index (BMI), smoking status, and beta-blocker use. For each variable, we tested normality and then applied the Mann–Whitney U test to compare the medians of 24 h entropy and symbolic dynamic parameters between groups. We then correlated entropy measures and symbolic metrics with time-domain and frequency-domain parameters. All statistical tests were two-sided, with a 95% confidence level (α = 0.05).

The Cosinor model is y(t) = M + A cos (ωt + φ) + ε (t), where ω = 24 h was applied to HRV standard parameters and symbolic dynamic parameters, where a more regular circadian modulation was observed. In contrast, entropy parameters were represented using a non-parametric approach to preserve the intrinsic complexity and irregular temporal structure of these measures. Although the Cosinor model is a standardized and widely used method for analyzing circadian rhythms, it has limitations in capturing complex temporal dynamics, specifically in signals characterized by non-stationary, multi-scale variability, and deviations from a sinusoidal pattern (Figure S4).

In the analyzed parameters modeled by the cosinor function, least-squares regression was used to estimate MESOR, amplitude, and acrophase. Linear mixed-effects models were applied to individual Cosinor parameters (MESOR and amplitude), including group, age, and sex as fixed effects and subjects as random effects. We obtained acrophases from individual cosinor fits and analyzed them using circular statistics; phase organization was assessed by the Rayleigh test. Group differences in acrophase distributions were evaluated by the Mardia–Watson–Wheeler test. All statistical analyses were performed using R (version 4.2.2), including the card, cosinor2, and nlme packages, and GraphPad Prism (version 8.0.0 for Windows).

### 2.9. Coordigram-Based Coordination and Entropy Analyses

Cardiorespiratory coordination was quantified as the percentage of coordinated heartbeats (%) computed over the full 24 h recording and hour-by-hour (0–23 h). Coordination was estimated using two temporal tolerance parameters (ε = 0.1 s and ε = 0.2 s). Group differences in the 24 h coordination percentage were assessed using a two-sided Mann–Whitney U test (α = 0.05). For hourly coordination profiles and hourly entropy indices, we performed Mann–Whitney U tests at each hour (control vs. heart failure). To account for multiple hourly comparisons, *p*-values were adjusted to control the false discovery rate (FDR) using the two-stage step-up procedure of Benjamini, Krieger, and Yekutieli, with the desired FDR (Q) = 5%. These hour-by-hour analyses were intended as structured exploratory comparisons of circadian profiles, aimed at identifying time windows of potential group separation rather than testing a single global hourly effect. Because hourly testing involves multiple comparisons across the 24 h cycle, these analyses were interpreted as structured exploratory comparisons of circadian profiles. Accordingly, the strongest emphasis was placed on patterns that persisted after FDR correction or appeared consistently across consecutive hours.

As part of this exploratory analysis, we examined the relationship between cardiorespiratory coordination (%) and PRQ timing complexity, FuzzEn_PRQ_, at the individual level using Spearman’s rank correlation (two-sided). For each participant, hourly values were averaged over three periods: (i) the entire 24 h profile; (ii) daytime (07:00–22:00); and (iii) nighttime (01:00–04:00). We calculated correlations for both coordination tolerances (ε = 0.1 s and ε = 0.2 s), considering all subjects together and separated by group (controls vs. HF). Ninety-five percent confidence intervals for Spearman’s ρ were derived through the non-parametric bootstrap resampling of subjects. Where applicable, uncertainty was summarized using bootstrap-based 95% confidence intervals, particularly for acrophase concentration differences and subject-level correlation analyses reported in the Supplementary Material.

## 3. Results

We studied 88 controls and 75 patients with HF; no differences were observed by sex or BMI (Table 1). Patients with HF had a mean left ventricular ejection fraction (LVEF) of 49% with a SD of 13%. Accordingly, 61% were classified as having preserved EF, 10% were classified as mildly reduced EF, and 29% were classified as having reduced EF. In addition, 61% of patients presented with concentric structural remodeling, consistent with systolic dysfunction. The sleep diary reported a mean sleep time of 22:00 and a mean wake time of 6:00. Controls were reported without abnormalities or cardiac remodeling on transthoracic echocardiography.

We present initial supportive evidence of reduced overall cardiac autonomic activity and information content on HF, as assessed by standard HRV and symbolic measures. This is followed by evidence of circadian chronodisruption, as indicated by cosinor-derived phase dispersion. We then share primary findings on CRC, measured as coordination (%) at ε = 0.1 s and ε = 0.2 s. Finally, we highlight entropy-based hour-of-day results, emphasizing the two measures that remained significant after FDR correction: early-night ApEn_BB_ (1:00–4:00) and daytime/evening FuzzEn_PRQ_ (≈7:00–22:00). Effect-size-oriented interpretation was supported by the direction, consistency, and temporal persistence of the observed patterns, while bootstrap 95% confidence intervals are provided in the supplementary analyses where applicable.

### 3.1. Supportive Evidence: Overall Cardiac Autonomic Alterations in Heart Failure

In the time-domain analysis, patients with heart failure (HF) exhibited significantly reduced heart rate variability compared with controls, as reflected by a lower SDNN (HF median 107.5; IQR 47 vs. controls median 136; IQR 41; Mann–Whitney U = 4528; df = 161; *p* < 0.001), cvNN (HF median 0.12; IQR 0.06 vs. controls median 0.17; IQR 0.05; Mann–Whitney U = 5116; df = 161; *p* < 0.001), SDANN1 (HF median 96.4; IQR 39.6 vs. controls median 126.2; IQR 46.8; Mann–Whitney U = 4708; df = 161; *p* < 0.001), SDANN5 (HF median 90.2; IQR 41.2 vs. controls median 120.5; IQR 47.4; Mann–Whitney U = 4770; df = 161; *p* < 0.001), and SDANN10 (HF median 88.2; IQR 40.7 vs. controls median 117; IQR 49.5; Mann–Whitney U = 4767; df = 161; *p* < 0.001).

In the frequency domain, patients with HF exhibit a characteristic pattern with overall autonomic HRV-related parameters appearing lower. Very-low-frequency power (VLF ms^2^) was significantly reduced in patients with HF (HF median 0.6; IQR 0.6 vs. controls median 0.9; IQR 1.0; Mann–Whitney U = 4403; df = 161; *p* < 0.001). Low-frequency power (LF ms^2^) was significantly reduced in patients with HF (HF median 0.13; IQR 0.3 vs. controls median 0.42; IQR 0.6; Mann–Whitney U = 4403; df = 161; *p* < 0.001). High-frequency power (HFp ms^2^) was significantly reduced in patients with HF (HF median 0.04; IQR 0.1 vs. controls median 0.07; IQR 0.09; Mann–Whitney U = 3977; df = 161; *p* = 0.02). Normalized low-frequency power (LFn) was also lower in the HF group (HF median 0.7; IQR 0.2 vs. controls median 0.9; IQR 0.1; Mann–Whitney U = 5043161; *p* < 0.001), whereas normalized high-frequency power (HFn) was higher in patients with HF (HF median 0.2; IQR 0.2 vs. controls median 0.1; IQR 0.1; Mann–Whitney U = 1557; df = 161). Accordingly, the LF/HF ratio was higher in controls (HF median 3.1; IQR 3.9 vs. controls median 6.2; IQR 5.5; Mann–Whitney U = 5043; df = 161; *p* < 0.001).

Multivariable linear regression models adjusted for age, sex, and beta-blocker use showed that several HRV metrics remained significantly different between healthy controls and patients with heart failure after false discovery rate correction (FDR) (Table S1). HF was independently associated with mean NN, pNN50, pNN100, pNNl50, LF, HF, LFn, and HFn (all adjusted *p* < 0.05).

Regarding nonlinear dynamics, significant differences were observed in Shannon entropy (HF median 3.0; IQR 0.5 vs. controls median 3.2; IQR 0.3; Mann–Whitney U = 4629; df = 161; *p* < 0.001), Rényi entropy (α = 0.25) (HF median 2.8; IQR 0.3 vs. controls median 2.9; IQR 0.2; Mann–Whitney U = 4227; df = 161; p = 0.002), Rényi entropy (α = 2) (HF median 2.8; IQR 0.3 vs. controls median 2.9; IQR 0.2; Mann–Whitney U = 4712; df = 161; *p* < 0.001) and Rényi entropy (α = 4) (HF median 2.8; IQR 0.3 vs. controls median 2.9; IQR 0.2; Mann–Whitney U = 4725; df = 161; *p* < 0.001). Overall, patients with HF exhibited lower entropy values, indicating lower uncertainty, decreased information content, and fewer rare recurrent patterns, consistent with a loss of dynamical complexity in cardiac autonomic regulation.

### 3.2. Circadian Chronodisruption in Symbolic Cardiac Dynamics in Heart Failure

**Figure 2:**
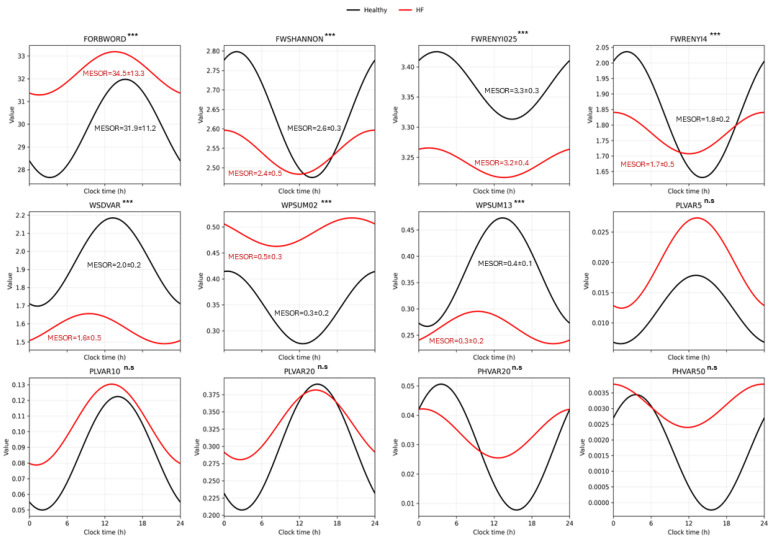
**Circadian distribution of symbolic dynamic metrics.** Black lines represent control subjects, and red lines represent patients with heart failure. Curves were fitted using a single-component cosinor model with a fixed 24 h period. Group comparisons of cosinor-derived parameters were performed using the Mann–Whitney U test. Amplitudes were significantly lower in patients with HF for Rényi entropy (α = 4), PHVAR20, and WPSUM13. MESOR is presented as Mdn ± IQR. *** indicates *p* < 0.001 for between-group differences in MESOR (Mann–Whitney U test). n.s indicates no significant between-group differences in MESOR.

Circadian rhythmicity differed from controls and patients with HF across entropy and symbolic dynamics metrics; significant differences were mainly observed in MESOR, reflecting lower oscillatory activity in the HF group (Figure 2 and Figure S2). Although mean acrophases did not differ significantly between groups, patients with HF exhibited significantly reduced phase concentration. The mean resultant length (R) in HF, compared with controls, indicated increased phase dispersion and circadian desynchronization across all evaluated metrics, suggesting chronodisruption in the HF group (See Table S1).

Mixed-effects models adjusted for age and sex found no significant group-by-sex effect on any circadian parameter. Age exerts an effect on MESOR and amplitude for: FORBWORD, FWSHANNON, FWRENYI25, FWRENYI4, WSDVAR, WPSUM13, PLVAR20, and PHVAR20. HF consistently affects symbolic metrics: WSDVAR, WPSUM02, WPSUM13, and PHVAR20. This suggests that aging exerts a global modulatory effect on circadian autonomic dynamics, while HF introduces additional, domain-specific alterations rather than a uniform shift across all the evaluated metrics (see Table S1).

### 3.3. Cardiorespiratory Coupling Is Altered in Patients with HF 

To quantify time- and tolerance-dependent remodeling of CRC in its event-timing coordination mode, we assessed the percentage of coordinated beats over the entire 24 h period and on an hour-by-hour basis.

#### 3.3.1. CRC During the 24 H

**Figure 3:**
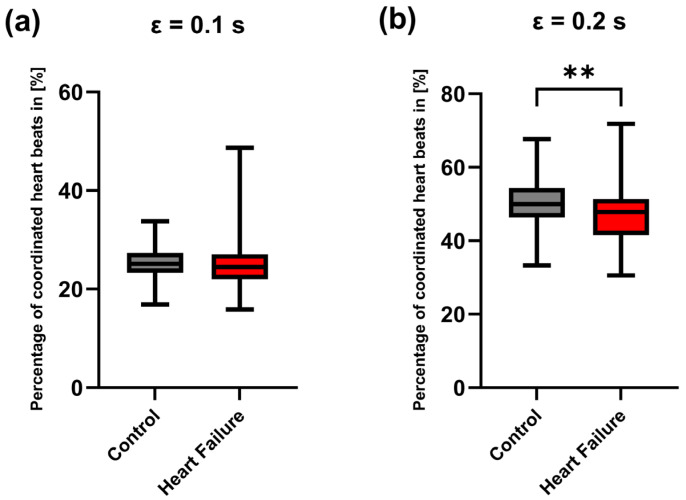
Twenty-four-hour cardiorespiratory coordination in control and heart failure. Box-and-whisker plots show the percentage of coordinated heartbeats (%) computed over the full 24 h recording for (**a**) ε = 0.1 s and (**b**) ε = 0.2 s. The center line indicates the median, the box spans the interquartile range (25th–75th percentiles), and the whiskers represent the minimum to maximum values. Group differences were assessed using a two-sided Mann–Whitney U test. ** indicates *p* < 0.01.

Across the full 24 h recordings, the percentage of coordinated heartbeats depended on the tolerance parameter ε. With stricter tolerance (ε = 0.1 s), control and heart failure showed comparable median coordination (Figure 3a). In contrast, with ε = 0.2 s, the control group exhibited a higher 24 h coordination percentage than the heart failure group (Mann–Whitney U test, *p* < 0.01) (Figure 3b).

**Figure 4:**
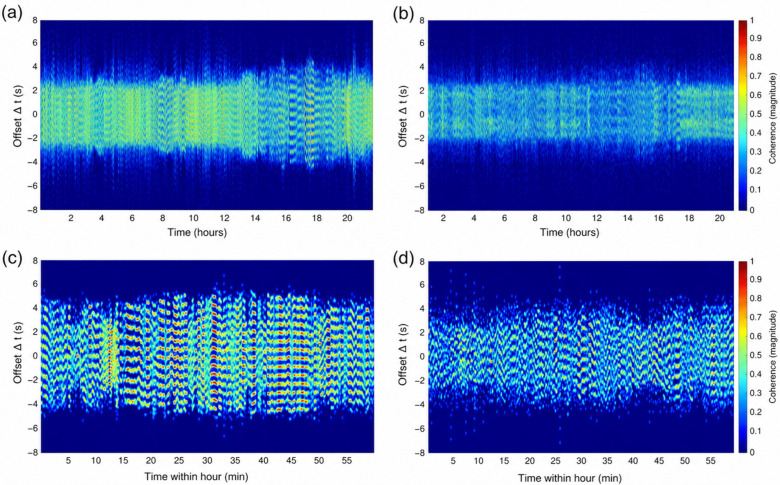
Representative coordigrams of cardiorespiratory coordination (CRC) in control and heart failure. Panels (**a**,**b**) show coordigrams for the full recording plotted across the day (time-of-day, 0–23 h). Panels (**c**,**d**) show 1 h coordigrams from the same respective participants as in (**a**,**b**), displayed as time within the hour (min). In each coordigram, the y-axis represents the time offset between cardiac and respiratory events (Δt, s), and color encodes the coordination probability (0–1; warmer colors indicate higher probability). Control examples are shown in (**a**,**c**), whereas examples of heart failure are shown in (**b**,**d**). The sign convention is such that Δt > 0 indicates cardiac events occurring after the respiratory reference event (respiration → heart), while Δt < 0 indicates cardiac events preceding it (heart → respiration). Horizontal bands/lines indicate preferred, stable delays (i.e., persistent coordination at specific Δt values).

Representative coordigrams illustrate qualitative differences in the coordination structure (Figure 4). In the control example, the 24 h coordigram (Figure 4a) shows a comparatively broader coordination ridge at around Δt ≈ 0 with noticeable time-varying modulation, and the within-hour view (Figure 4c) reveals pronounced horizontal banding that drifts over time, consistent with dynamically changing preferred delays. In the example of heart failure, coordination appears more confined around a narrower, relatively stable ridge at specific offsets in the 24 h view (Figure 4b), whereas the 1 h coordigram (Figure 4d) is more diffuse with less sharply defined bands, suggesting a weaker or less organized within-hour delay structure.

#### 3.3.2. CRC Hour Profile

**Figure 5:**
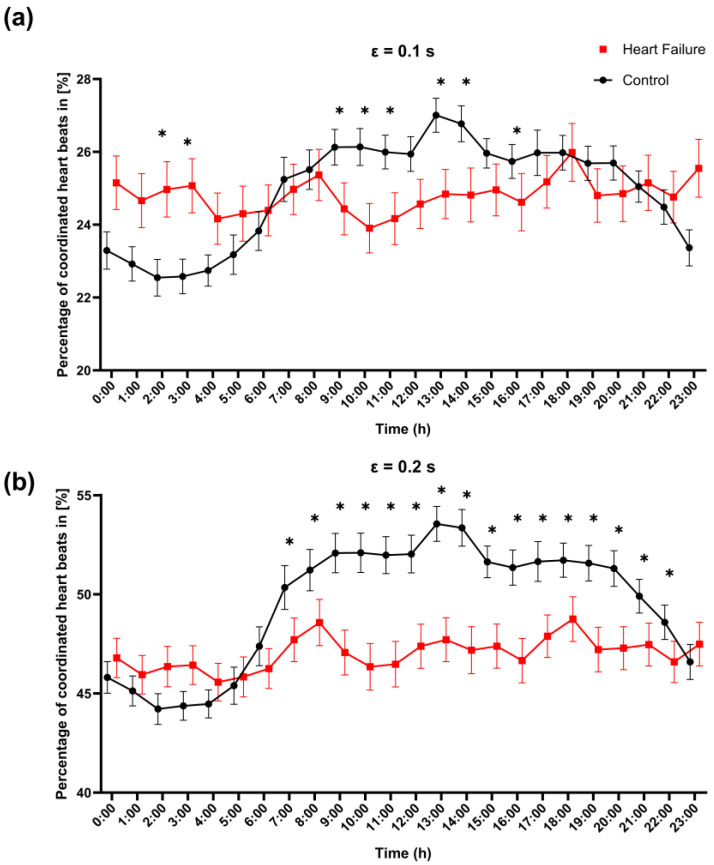
Hour-of-day profile of cardiorespiratory coordination in controls and patients with heart failure. Hourly values of the percentage of coordinated heartbeats (%) are shown across the 24 h day for (**a**) ε = 0.1 s and (**b**) ε = 0.2 s (control: black circles; heart failure: red squares). Error bars indicate SEM. At each hour (0–23 h), groups were compared using a two-sided Mann–Whitney U test, and *p*-values were adjusted for multiple comparisons across hours using FDR (Benjamini, Krieger, and Yekutieli two-stage step-up) with Q = 5%. * indicates FDR-adjusted significance (*q* < 0.05) at the corresponding hour.

Hourly analyses further indicated a marked hour-of-day modulation of coordination (Figure 5). For ε = 0.1 s, between-group differences were hour-dependent: heart failure showed higher coordination during the early-night window, specifically at 2:00 and 3:00, whereas the control exceeded heart failure during the late morning (9:00–11:00) and early afternoon (13:00–14:00), with an additional difference at 16:00 (starred hours represent an FDR-adjusted value of q < 0.05; Figure 5a). For ε = 0.2 s, the separation between groups became more sustained: controls displayed a pronounced daytime/evening elevation with higher coordination than patients with heart failure across extended consecutive hours (approximately 7:00–15:00 and 17:00–22:00; q < 0.05), with a smaller late-hour difference at 23:00 (Figure 5b).

### 3.4. Primary Entropy Findings: Hour-of-Day Dynamics of RR, BB, and PRQ

To complement the event-timing view of CRC provided by coordination (%), we also evaluated pattern-based CRC indices derived from RR–BB dynamics, alongside single-series entropy in RR, BB, and PRQ. Following FDR correction over hours, two entropy-based patterns emerged: respiratory ApEn_BB_ was elevated in HF during early night (1:00–4:00), and FuzzEn_PRQ_ was higher in controls throughout the daytime and early evening (≈7:00–22:00). Because the respiratory timing difference was observed for ApEnBB, while SampEnBB and FuzzEnBB showed overlapping profiles without FDR-significant hourly differences, this result should be interpreted cautiously as estimator-dependent. By contrast, the FuzzEnPRQ pattern appeared more sustained across consecutive daytime and early-evening hours. Other entropy measures (RR-based and cross-entropy RR–BB) showed mostly overlapping profiles and did not survive hourly FDR correction, suggesting that coordination (%) was more sensitive than entropy-based timing irregularity at the hourly scale. Accordingly, isolated hourly tendencies that did not survive FDR correction were not interpreted as robust group effects.

**Figure 6:**
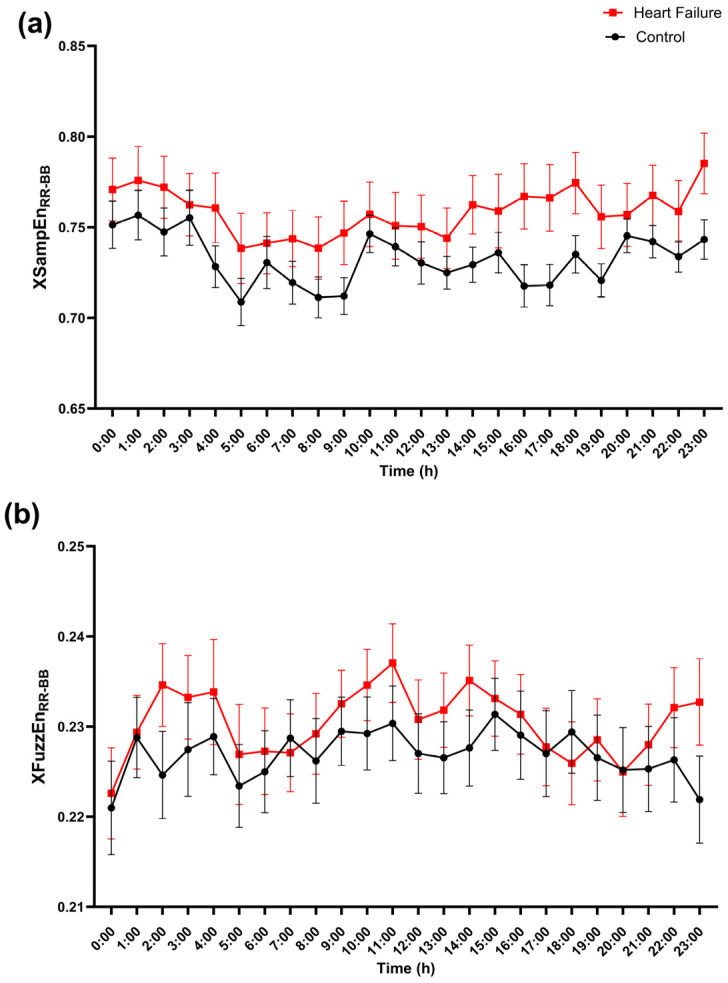
Hour-of-day profile of cross-entropy between cardiac and respiratory timing (RR–BB). Hourly values of (**a**) cross-sample entropy (XSampEn_RR–BB_) and (**b**) cross-fuzzy entropy (XFuzzEn_RR–BB_) computed between RR and breath-to-breath (BB) interval series are shown across the 24 h day (0–23 h) for controls (black circles) and patients with heart failure (red squares). Points represent mean values, and error bars indicate SEM. At each hour, groups were compared using a two-sided Mann–Whitney U test, and *p*-values were adjusted for multiple comparisons across hours using FDR control with the two-stage step-up procedure of Benjamini, Krieger, and Yekutieli (Q = 5%).

**Figure 7:**
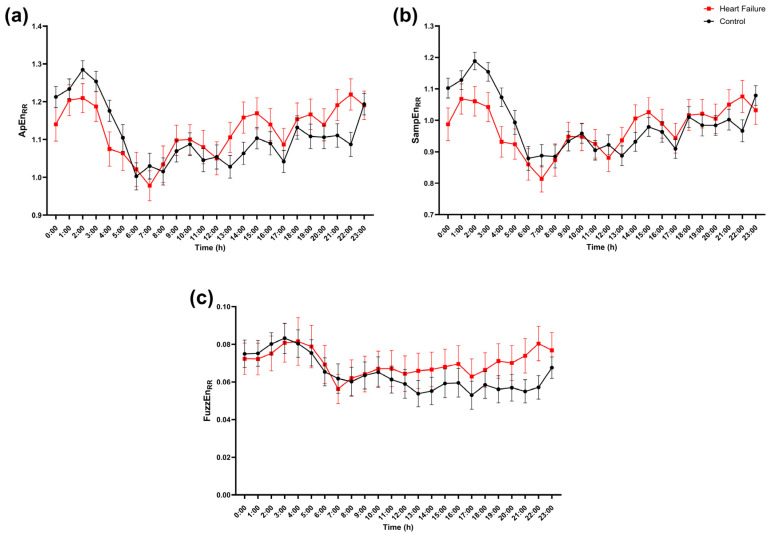
Hour-of-day profile of cardiac timing entropy (RR series). Hourly values of (**a**) approximate entropy (ApEn_RR_), (**b**) sample entropy (SampEn_RR_), and (**c**) fuzzy entropy (FuzzEn_RR_) computed from the RR interval series are shown across the 24 h day (0–23 h) for controls (black circles) and patients with heart failure (red squares). Points represent mean values, and error bars indicate SEM. For each hour, groups were compared using a two-sided Mann–Whitney U test, and *p*-values were adjusted for multiple hourly comparisons using FDR correction (Benjamini, Krieger, and Yekutieli two-stage step-up; Q = 5%).

**Figure 8:**
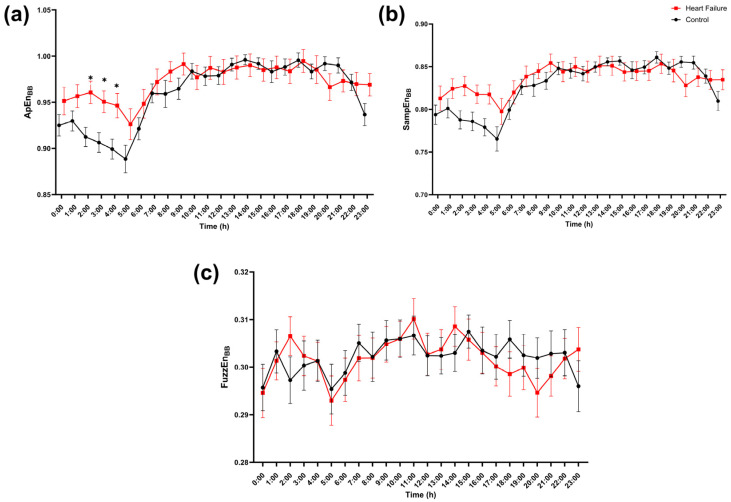
Hour-of-day profile of respiratory timing entropy (BB series). Hourly values of (**a**) approximate entropy (ApEn_BB_), (**b**) sample entropy (SampEn_BB_), and (**c**) fuzzy entropy (FuzzEn_BB_) computed from the breath-to-breath (BB) interval series are shown across the 24 h day (0–23 h) for controls (black circles) and patients with heart failure (red squares). Points represent mean values, and error bars indicate SEM. At each hour, groups were compared using a two-sided Mann–Whitney U test, with FDR correction across the 24 h tests using the Benjamini–Krieger–Yekutieli two-stage step-up procedure (Q = 5%). * indicates FDR-adjusted significance (*q* < 0.05) at the corresponding hour.

**Figure 9:**
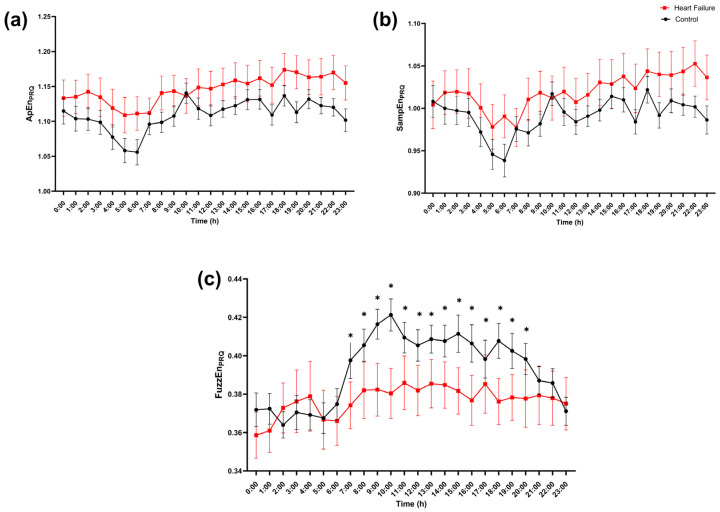
Hour-of-day profile of pulse–respiration quotient entropy (PRQ series). Hourly values of (**a**) approximate entropy (ApEn_PRQ_), (**b**) sample entropy (SampEn_PRQ_), and (**c**) fuzzy entropy (FuzzEn_PRQ_) computed from the pulse–respiration quotient (PRQ) time series are shown across the 24 h day (0–23 h) for controls (black circles) and patients with heart failure (red squares). Points represent mean values, and error bars indicate SEM. Group differences at each hour were tested with a two-sided Mann–Whitney U test and corrected for multiple comparisons across hours using FDR (Benjamini, Krieger, and Yekutieli two-stage step-up; Q = 5%). * indicates FDR-adjusted significance (*q* < 0.05) at the corresponding hour.

Consistent with the hourly coordination profiles, entropy-based indices showed only modest group separation when examined across the 24 h day (Figure 6, Figure 7, Figure 8 and Figure 9). Cross-entropy between RR and BB displayed a small but consistent upward shift in heart failure: XSampEn_RR–BB_ was generally higher in heart failure than in controls across most hours (Figure 6a), and XFuzzEn_RR–BB_ showed a similar tendency with substantial overlap between groups (Figure 6b). However, no individual hours survived FDR correction for these cross-entropy measures.

In ambulatory 24 h recordings, coordination (%) derived from event timing was more sensitive than cross-entropy to detect group differences after hourly multiple-comparison control, likely because cross-entropy metrics are more affected by within-hour behavioral heterogeneity (posture, speech, and meals) and nonstationarity.

For single-series cardiac timing entropy (RR), both groups exhibited broadly similar hour-of-day trajectories with overlapping confidence intervals (Figure 7). ApEn_RR_ and SampEn_RR_ showed parallel diurnal modulation without FDR-significant hourly differences (Figure 7a,b). FuzzEn_RR_ suggested a mild elevation in heart failure during the later part of the day, yet this separation likewise did not reach FDR significance (Figure 7c).

Group differences were more localized for respiratory timing (BB) (Figure 8). The corresponding hour-of-day respiratory rate profile is shown in Figure S3. ApEn_BB_ was higher in heart failure during the interval of the early night (1:00–4:00), corresponding to hours that remained significant after adjustment for FDR (Figure 8a). In contrast, SampEn_BB_ and FuzzEn_BB_ showed substantial overlap between groups across the day with no FDR-significant hourly differences (Figure 8b,c), indicating that the respiratory timing effect was not equally expressed across entropy estimators.

Finally, PRQ-derived entropy revealed the clearest and most sustained hour-specific separation in FuzzEn_PRQ_ (Figure 9). ApEn_PRQ_ and SampEn_PRQ_ showed only small between-group differences without FDR-significant hours (Figure 9a,b). In contrast, FuzzEn_PRQ_ was higher in controls than in heart failure across the daytime and early evening hours (approximately 7:00–22:00), with multiple consecutive hours reaching FDR-adjusted significance (Figure 9c). Overall, after correction for multiple hourly comparisons, significant differences were restricted to early-night ApEn_BB_ and the daytime/evening elevation of FuzzEn_PRQ_ in controls, while cross-entropy and RR-based entropy measures mainly showed non-significant trends.

In exploratory subject-level analyses, CRC and FuzzEn_PRQ_ showed a weak positive association during daytime (ε = 0.2), driven mainly by the HF group, whereas 24 h and nocturnal averages showed no meaningful association (Table S3).

## 4. Discussion

Heart failure is a heterogeneous syndrome characterized by underlying hemodynamic, autonomic, neurohumoral, and circadian alterations, a chronic progressive course, and multisystem involvement. Recent advances in diagnosis and treatment have improved risk stratification and therapeutic options. However, HF remains a highly prevalent condition worldwide, often associated with delayed diagnosis and substantial mortality [45,46]. The circadian rhythmicity of cardiac and respiratory markers has recently gained attention for its potential role in early diagnosis, risk stratification, and therapeutic guidance in patients with heart failure [47,48]. Accordingly, in this work, we analyzed the circadian dynamics of cardiac autonomic function and CRC, with event-timing coordination as the primary operational measure, using entropy-based, symbolic dynamics, and coordination metrics to explore early candidate biomarkers. We found that patients with HF exhibit marked alterations in 24 h cardiac autonomic dynamics; cardiorespiratory coordination is impaired with a clear time-of-day modulation, and the pulse–respiration quotient emerges as a promising marker for characterizing daytime CRC integration. Understanding cardiorespiratory complexity across the circadian cycle may provide a powerful framework for uncovering hidden temporal patterns in cardiac signals and elucidating the interrelated mechanisms linking the autonomic, respiratory, and circadian systems in the pathophysiology of heart failure.

Patients with HF exhibited altered 24 h cardiac autonomic dynamics characterized by reduced flexibility and complexity. Across the circadian cycle, patients with HF showed markedly reduced global HRV and lower Shannon and Rényi entropies of RR dynamics. VLF power, considered a marker of circadian and neurohumoral modulation, was also reduced in patients with HF, consistent with the autonomic and neurohumoral disturbances described in heart failure [49]. These findings highlight loss of complexity and richness in cardiac autonomic activity in HF, consistent with impaired autonomic regulation, neurohumoral dysregulation, and inflammatory activation, as well as a reduced repertoire of physiological responses and reduced information generation across the circadian cycle [50,51,52]. Moreover, HF exerts an effect on circadian activity consistent with chronodisruption: cosinor analyses indicated that group differences were mainly driven by reduced rhythmic strength, as evidenced by changes in MESOR and amplitude across entropy and symbolic dynamics indices. Critically, patients with HF exhibited marked acrophase dispersion, indicating impaired circadian phase coordination and internal desynchronization. Temporal disruptions in oscillatory activity and acrophase consistency may be related to structural, metabolic, and molecular remodeling processes that influence peripheral oscillator function by altering clock gene regulation and post-translational mechanisms [41,53,54].

Additionally, the alteration of CRC in heart failure indicates reduced cardiorespiratory coordination with a time-of-day dependence. Building on the framework that cardiorespiratory interactions comprise respiratory sinus arrhythmia, heartbeat–inspiratory-onset synchronization, and respiratory stroke volume synchronization, with proposed roles in stabilizing systemic flow and improving cardiac efficiency, our observation of a tolerance- and time-of-day-dependent CRC reorganization in HF is consistent with a shift in the expression of these oscillatory mechanisms across daily states [55]. Additionally, regarding lagged joint symbolic dynamics, previous evidence shows that heart period and respiration remain coupled during both spontaneous and controlled breathing, while controlled breathing can redistribute coupling across time scales without a preferred lag. From a systems perspective, impaired acrophase coherence and tolerance-dependent CRC alterations may reflect weakened coupling between central and peripheral oscillators, as evidenced by reduced flexibility in autonomic–respiratory phase coordination. HF-related remodeling becomes most evident when CRC is examined across broader timing criteria and within an explicit chronobiological context, rather than at a single interaction scale [56].

In this sense, the time-of-day dependence suggests that CRC alterations in HF are strongly state-dependent, modulated by autonomic challenges. This is consistent with coordigram-based evidence of obstructive sleep apnea, which shows that CRC is more frequent during sleep-related breathing instability and persists after these events. Even though these post-event epochs are characterized by high autonomic stress, they challenge the view that spontaneous CRC is limited to relaxed conditions [57,58]. Together, these findings support the idea that sleep–wake state and transient ventilatory/autonomic shifts can concentrate differences in CRC in specific nocturnal versus daytime windows; this hypothesis must be studied using PSG or validated respiratory sensors [59]. In this context, the nocturnal increase in ApEnBB in HF is compatible with a more irregular breathing pattern during sleep, since approximate entropy of respiratory movement has been shown to vary across wakefulness and sleep stages [56,57,58,60]. At the same time, because this effect was not consistently reproduced by SampEnBB or FuzzEnBB, it should be interpreted cautiously as estimator-dependent rather than as a stand-alone robust marker. Because mean respiratory rate and respiratory timing irregularity capture different properties, we report the hourly respiratory rate profile descriptively in Figure S3, while interpreting ApEn_BB_ as an index of timing irregularity rather than rate alone.

A central contribution of this study is that PRQ-based fuzzy entropy (FuzzEn_PRQ_) emerged as the most consistent discriminator of daytime cardiorespiratory integration at the hourly scale. Importantly, prior work directly applying entropy to PRQ indicates that PRQ entropy is responsive to interventions that deliberately reshape cardiorespiratory organization [61]. For example, induced relaxation/diaphragmatic breathing has been reported to reduce PRQ irregularity (lower SampEnPRQ and FuzzEnPRQ), together with changes in PRQ level and variability, is con-sistent with a more stereotyped breathing-driven pattern in the cardiac-to-respiratory ratio. However, a reduction in FuzzEnPRQ does not have a single physiological meaning by itself. Its interpretation depends on the context in which it occurs and on accompanying physiological markers. In intervention studies, lower PRQ entropy appears under externally guided conditions designed to promote organized cardiorespiratory patterning. In contrast, in the present study, lower daytime FuzzEnPRQ was observed spontaneously in HF during ambulatory daily-life conditions and together with lower HRV, reduced information content in RR dynamics, altered CRC, and circadian chronodisruption. In this setting, we interpret lower FuzzEnPRQ not as an isolated marker of beneficial stabilization, but as part of a broader pattern of reduced adaptive flexibility. Accordingly, FuzzEnPRQ alone is not sufficient to distinguish adaptive stabilization from maladaptive rigidity, and its interpretation should be supported by additional descriptors such as PRQ level and variability, RR and BB entropy profiles, coordination measures, respiratory state, and clinical context.

Consistent with this heterogeneity, Radovanović et al. reported that patients with HF show reduced or absent RR–respiration coherence and diminished bidirectional causality, yet cross-sample entropy could indicate higher RR–respiration synchrony in the subgroup with ventricular extrasystoles [16]. We cite this study to acknowledge that arrhythmic subgroups may show metric-specific behavior. However, this possibility was not directly evaluated in the present cohort, since recordings with high ectopy burden were excluded, and the analyses were restricted to artifact-controlled windows. Therefore, our findings should be interpreted as reflecting cardiorespiratory interaction patterns in the post-quality-control HF cohort, rather than as evidence of arrhythmia-specific effects. In addition, the measures applied here capture coordination and shared temporal structure, not directed causality itself.

The comparatively weaker separation observed for cross-entropy between RR and BB after multiple-comparison control also fits with recent methodological work showing that different “cross-” families (cross-entropy, cross-unpredictability, and state-space correspondence) can vary in sensitivity depending on data length, nonstationarity, and how the interaction manifests (tight deterministic locking vs. intermittency vs. lagged coordination) [62]. In day-long ambulatory conditions, respiration and heart period are influenced by posture shifts, speech, meals, and micro-arousals (Figure S2); such heterogeneity may dilute hour-specific effects for cross-entropy metrics. A direct test of whether these indices become more sensitive under more homogeneous conditions would require objective state annotation or predefined controlled resting segments, which were not available in the present dataset. Accordingly, this interpretation should be viewed as a plausible methodological explanation rather than as a result directly demonstrated here. Future studies incorporating more explicit control of behavioral state, as well as directionality and scale, may help clarify this issue [63].

**Figure 10:**
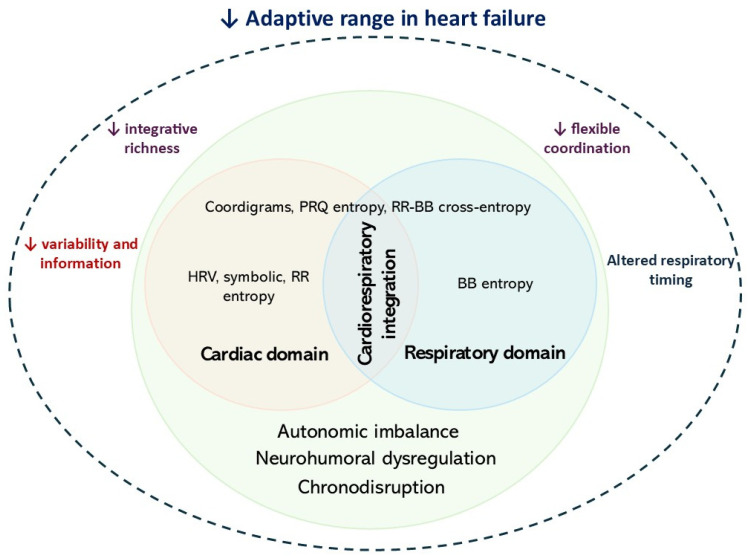
Adaptive range framework in circadian and temporal patterns in HF. Heart failure (HF) is associated with multilevel alterations that underlie autonomic, neurohumoral, and circadian mechanisms. At the cardiac level, patients with HF exhibited reduced global HRV and lower information content in RR dynamics, consistent with a narrower autonomic repertoire and diminished capacity to generate physiological variability. Circadian analyses additionally indicated chronodisruption, primarily expressed as reduced rhythmic strength and increased inter-individual variability in acrophase, rather than a uniform phase shift. At the cardiorespiratory integrative level, alterations in cardiorespiratory coupling (CRC) were not uniform but depended on both time of day and coordination tolerance. Differences between groups became more evident when coordination was assessed using broader temporal tolerances and at an hourly resolution, suggesting that HF is characterized by impaired flexible coordination under physiologically variable conditions rather than by a complete loss of strict synchronization. In entropy analyses, RR–BB cross-entropy did not manifest consistent hour-specific differences, whereas more robust signal-specific patterns arose, including increased nocturnal respiratory timing irregularity (ApEnBB) and reduced daytime PRQ-based integrative complexity (FuzzEnPRQ). Colors indicated the evaluated domains and levels of analysis (red: cardiac, blue: respiratory, purple: cardiorespiratory integration, green: systemic level). ↓ indicates diminished function/activity.

An integrated interpretation of these findings suggests that heart failure constrains state-dependent autonomic flexibility and reshapes cardiorespiratory regulation across several complementary levels. Importantly, the HRV, symbolic, entropy, and coordination metrics employed in this study represent complementary descriptors of different physiological domains and time scales, including autonomic modulation, temporal organization, respiratory dynamics, and integrative cardiorespiratory coordination. In this framework, reduced global HRV and lower RR-based information measures reflect a narrower autonomic repertoire and reduced information generation; altered symbolic and circadian indices reflect weaker temporal organization and chronodisruption; coordination percentages derived from the coordigram quantify how tightly cardiac timing remains organized relative to respiration across different timing tolerances; ApEnBB reflects respiratory timing irregularity; and FuzzEnPRQ reflects the richness of the integrated pulse–respiration relationship. From this perspective, the HF phenotype is interpreted as more constrained or rigid, not because every metric changes in the same direction, but because several complementary indices converge toward a reduced adaptive range, diminished integrative richness, and less flexible coordination across the day. This interpretation suggests a shared higher-level pattern that may arise from autonomic imbalance, neurohumoral dysregulation, and circadian desynchronization, even though the individual metrics probe different temporal scales and signal domains (Figure 10).

Importantly, coordination tolerance (ε) provides a scale-dependent lens: at ε = 0.1 s, the metric emphasizes very strict timing coincidence, whereas at ε = 0.2 s, it evaluates coordination within a broader timing window. The clearer group separation at ε = 0.2 s suggests that HF is more consistently associated with reduced coordination when the interaction is assessed within a wider temporal window and may be more compatible with ambulatory physiological variability, although its specific clinical and autonomic correlates remain to be established. The observation that HF-related differences occur primarily at larger ε suggests that heart failure is characterized not by a complete loss of coordination, but by a reduction in adaptive flexibility. Together, these data support the interpretation of HF as a state of multilevel physiological constraints involving impaired autonomic modulation, reduced integrative richness, and circadian desynchronization. Chronodisruption and temporal alterations of cardiorespiratory activity in HF should bring clinicians’ attention to critical time-of-day windows associated with increased vulnerability to adverse outcomes, with potential implications for risk stratification, therapeutic timing, and personalized management. In HF, information dynamics in ECG signals exhibited characteristic patterns during the circadian cycle, with lower complexity, fluctuations, and disturbed acrophase phenotypes. Cardiac alterations impact the respiratory system, leading to the stable and rigid cardiorespiratory coordination observed in HF. This may reflect reduced adaptive capacity of the regulatory system and may represent a marker of physiological dysregulation; however, any prognostic interpretation should be considered preliminary pending longitudinal validation. In addition, the higher coupling observed in HF during sleep may point to altered cardiorespiratory interaction patterns and possible sleep-related disturbances that remain clinically unrecognized and warrant further attention.

## 5. Limitations

HF is a complex and heterogeneous clinical syndrome encompassing diverse sociodemographic factors, pathophysiological mechanisms, phenotypes, disease severity, and dynamic clinical trajectories. This heterogeneity may limit the interpretation of time-scale-dependent analyses such as circadian assessments. In addition, the widespread use of medications, particularly β-blockers, which modulate heart rate, autonomic tone, and circadian variability, represents a potential confounding factor that may influence the observed dynamics. Nevertheless, consistent circadian patterns in autonomic regulation and cardiac electrical activity were identified, which may help characterize temporal vulnerability windows and the chronobiological organization of cardiorespiratory control in HF.

Regarding potential circadian confounders, physical activity is known to significantly influence cardiac autonomic modulation and heart rate dynamics across the day. The relative contribution of activity-related effects versus intrinsic circadian regulation remains incompletely understood and warrants further investigation. In the present study, objective measures such as actigraphy are unavailable, limiting the ability to directly quantify activity levels. Likewise, because objective sleep-state or activity annotations were not available, we could not isolate physiologically homogeneous sleep or resting segments for a dedicated reanalysis of cross-entropy sensitivity. To partially address this limitation, we analyzed circadian profiles of mean heart rate, including minimum and maximum values, as indirect proxies of physiological state. Additionally, nighttime periods were considered more controlled conditions, reducing behavioral variability and allowing more reliable between-group comparisons.

We acknowledge that interpolation may influence entropy estimates; therefore, these measures should be interpreted as reflecting the temporal organization of the uniformly sampled interval representations rather than being representative of the raw event series themselves. Because the present analyses were based on coordination and entropy-derived shared structures, future studies using directed interaction methods will be needed to determine whether these alterations also involve changes in causal cardiorespiratory influence.

Although recordings with high ectopic burden were excluded and the analysis was restricted to artifact-controlled windows, residual low-burden ectopy may still influence some RR-based, entropy-derived, and coordination-based indices. Accordingly, the present findings should be interpreted as characterizing the post-quality-control cohort rather than as an arrhythmia-stratified analysis, and the specific impact of residual ectopy remains an open question for future work. In addition, we did not perform a formal sensitivity analysis of the kernel density bandwidth used for coordigram smoothing; therefore, the KDE-based maps should be interpreted primarily as descriptive visualizations, whereas the main quantitative CRC results rely on the epsilon-strip coordination indices.

Finally, because no reference respiratory signal was recorded simultaneously, the reliability of EDR could not be validated directly in this cohort. This is particularly relevant in HF, where abnormal breathing patterns may affect the performance of ECG-derived respiratory surrogates [32].

## 6. Conclusions

Heart failure was associated with changes in both cardiac autonomic regulation and cardiorespiratory coupling over the 24 h day. At the cardiac level, patients with HF showed lower overall HRV and reduced information content in RR dynamics, suggesting a narrower range of autonomic modulation. Circadian analyses pointed to chronodisruption, which was expressed mainly as weaker rhythmic patterns and greater variability in timing across individuals, rather than a consistent shift in the rhythm. When we examined the integrative level, differences in CRC were not uniform: variations depended on the timing tolerance and the hour of day, and the separation between groups became clearer when coordination was assessed with a broader tolerance and analyzed hourly—highlighting specific windows in which coupling appears most affected. In the entropy analyses, RR–BB cross-entropy did not show any hour-specific differences. Instead, the most consistent signal-specific signatures were a higher nocturnal irregularity in respiratory timing (ApEn_BB_) in HF and a lower daytime/early-evening PRQ-integrated timing complexity (FuzzEn_PRQ_) compared with the controls. A higher early-night ApEnBB in HF was also observed, although this respiratory timing result should be interpreted more cautiously because it was not reproduced by SampEnBB or FuzzEnBB. Thus, the interpretation of lower FuzzEnPRQ should be made in conjunction with the broader autonomic and cardiorespiratory profile, since reduced PRQ entropy may reflect either adaptive stabilization or maladaptive rigidity depending on physiological context.

Overall, the HRV, symbolic, entropy, and coordination metrics used in this study should be interpreted as complementary descriptors of autonomic repertoire, temporal organization, respiratory timing irregularity, integrative cardiorespiratory richness, and coordination flexibility, not as equivalent markers of a single physiological property. Taken together, these findings support time-structured remodeling of cardiorespiratory dynamics in HF and point to the value of future studies that combine circadian profiling with careful characterization of sleep-related breathing instability and rhythm phenotypes to better understand mechanisms and clinical relevance.

## Figures and Tables

**Table 1:** Baseline characteristics between groups.

	Control (*n* = 88)	Heart Failure (*n* = 94)	*p*
Age (years: median–IQR)	47.5–13	62–10	<0.001 ^£^
Females (*n*–%)	45–51.1%	47–50%	0.878 ^Ş^
White ethnicity (*n*–%)	71–82.5%	70–81.3%	0.998
BMI (median–IQR)	24.7–4.47	24.22–2.32	0.96 ^£^
Self-reported smokers (*n*–%)	34–39.5%	10–11.6%	0.005 ^Ş^
Hypertension (*n*–%)	5–4.3%	43–50%	<0.001 ^Ş^

^Ş^ χ^2^ test. SD = standard deviation. ^£^ Mann–Whitney test. IQR = interquartile range.

## Data Availability

The datasets generated and/or analyzed during the current study are available from the corresponding author upon reasonable request due to privacy and ethical restrictions related to participant confidentiality.

## Supplementary Materials

The following supporting information can be downloaded at https://www.mdpi.com/article/10.3390/e28050524/s1. Figure S1. Flowchart selection of controls and HF patients. Figure S2. Hour-of-day profile of heart rate. Figure S3. Hour-of-day profile of respiratory rate in control and heart failure. Figure S4. Example of Cosinor modeling on symbolic dynamics indices. Table S1. Multivariate regression analysis of heart rate variability and entropy-related metrics: effects of group, age, sex, and beta-blocker treatment. Table S2. Acrophase concentration test. Table S3. Subject-level Spearman correlation between coordination percentage and FuzzEnPRQ.

## Abbreviations

The following abbreviations are used in this manuscript:

HF	Heart failure
ECG	Electrocardiogram
CRC	Cardiorespiratory coupling
HRV	Heart rate variability
EDR	ECG-derived respiration
BB	Breath-to-breath interval
RR	RR interval
PRQ	Pulse–respiration quotient
ApEn	Approximate entropy
SampEn	Sample entropy
FuzzEn	Fuzzy entropy
X-SampEn	Cross-sample entropy
X-FuzzEn	Cross-fuzzy entropy
MESOR	Midline estimating statistic of rhythm
FEVI (LVEF)	Left ventricular ejection fraction
SDNN	Standard deviation of normal-to-normal intervals
pNN	Percentage of successive normal-to-normal NN intervals that differ by more than a specific threshold (50, 100, 200 ms)
pNNl	Percentage of successive normal-to-normal NN intervals that differ less than a specific threshold (10, 20, 30, 50 ms)
cvNN	Coefficient of variation in normal-to-normal intervals
SDANN1	Standard deviation of the averages of NN intervals in 1 min segments
SDANN10	Standard deviation of the averages of NN intervals in 10 min segments
VLF	Very low frequency
HFp	High-frequency power
HFn	Normalized high-frequency power
LFn	Normalized low-frequency power
FORBWORD	Forbidden words (symbolic dynamics index)
FWSHANNON	Shannon entropy of forbidden words
FWRENYI25	Rényi entropy (α = 0.25) of forbidden words
FWRENYI4	Rényi entropy (α = 4) of forbidden words
WSDVAR	Word symbolic dynamics variance
WPSUM13	Percentage of symbolic patterns of classes 1 and 3
PLVAR20	Percentage of low-variability symbolic patterns (class 20)
PHVAR20	Percentage of high-variability symbolic patterns (class 20)
LF/HF	Low-frequency to high-frequency ratio
SEM	Standard error of the mean
FDR	False discovery rate
