# Supplementary material for: Temporal Properties of Cardiorespiratory Coupling in Patients with Heart Failure During the Circadian Cycle
Source: Entropy (Basel). 2026 May 6;28(5):524. doi: 10.3390/e28050524 (PMC13205341; doi:10.3390/e28050524)
Supplement: Supplementary file 1 [file entropy-28-00524-s001.zip › entropy-4150519-supplementary-Figures.pdf]

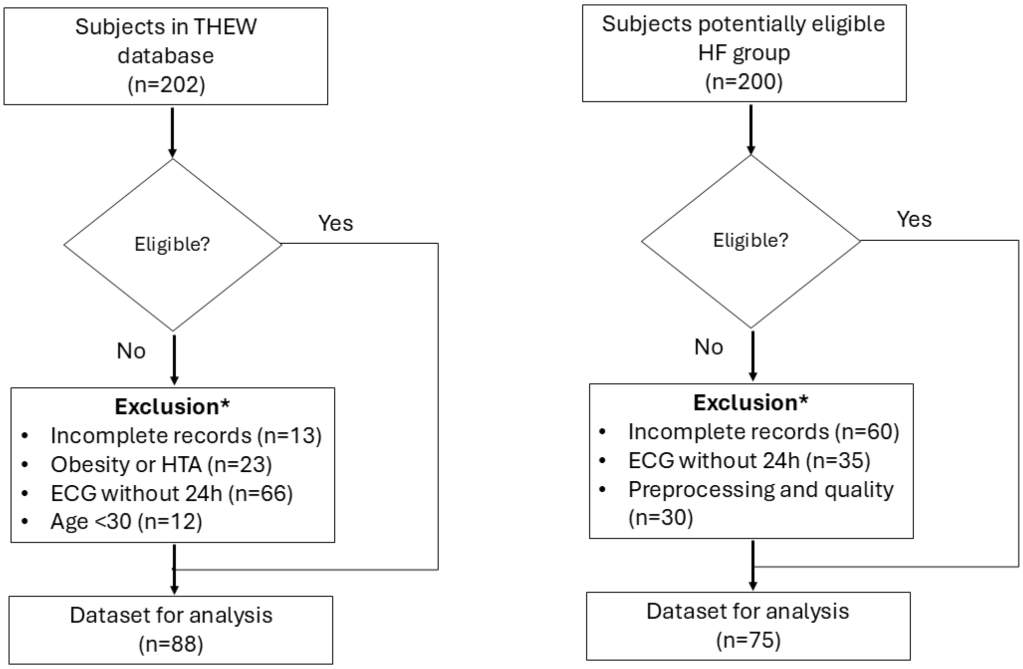

Figure S1. Flowchart selection of controls and HF patients.

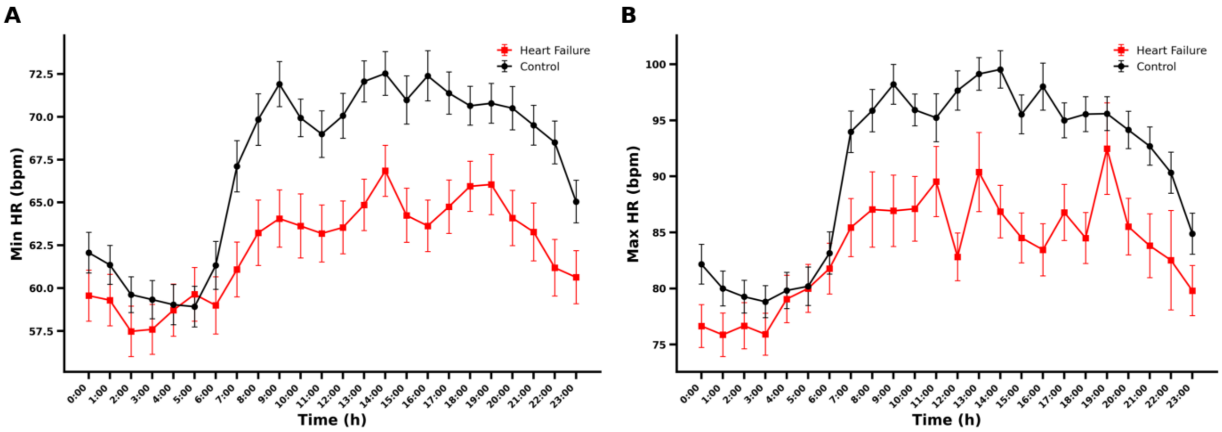

Figure S2. Hour-of-day profile of heart rate. Hourly heart rate profiles in controls and patients with heart failure. (A) Minimum heart rate (Min HR) by hour. (B) Maximum heart rate (Max HR) by hour. Values are shown as mean ± SEM for each hour of the day. Heart failure is represented by red squares and controls by black circles

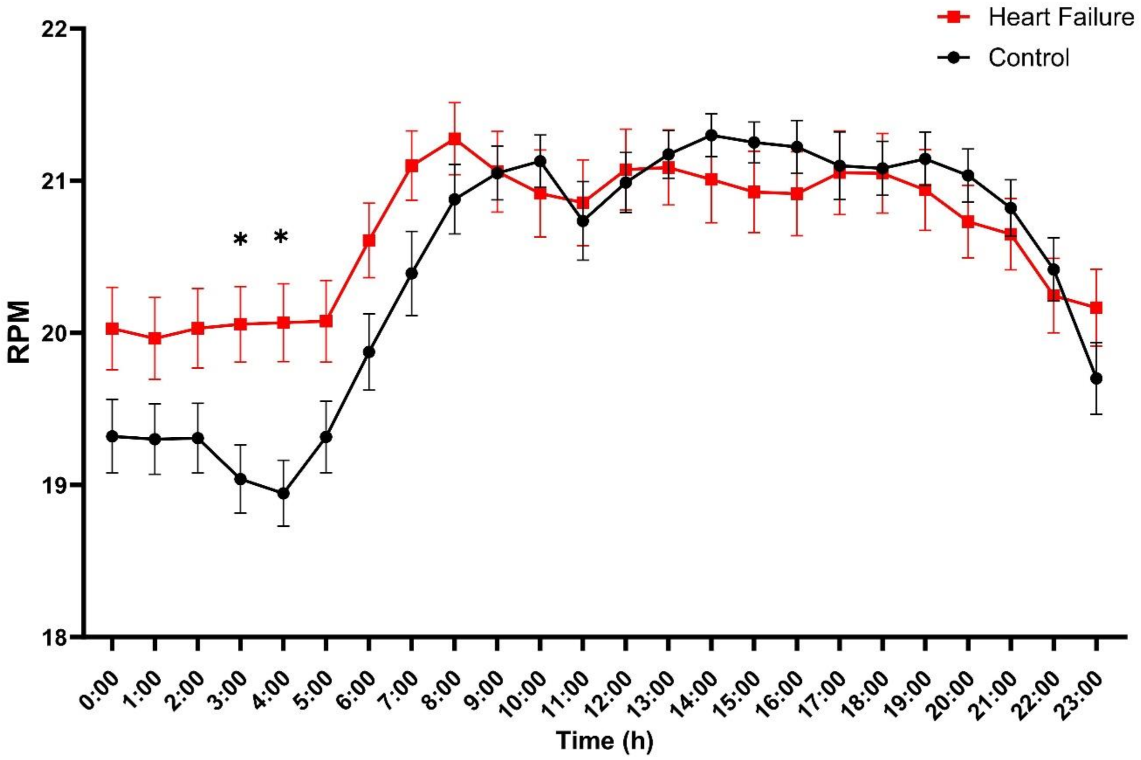

Figure S3. Hour-of-day profile of respiratory rate in control and heart failure. Hourly respiratory rate (RPM) derived from EDR-based breath-to-breath (BB) intervals (RPM = 60/BB) is shown across the 24-h day (0–23 h) for control (black circles) and heart failure (red squares). Points represent mean values and error bars indicate SEM. At each hour, groups were compared using a two-sided Mann–Whitney U test, and p-values were adjusted for multiple comparisons across hours using FDR control with the two-stage step-up procedure of Benjamini, Krieger, and Yekutieli ($Q = 5\%$). * indicates FDR-adjusted significance ($q < 0.05$) at the corresponding hour.

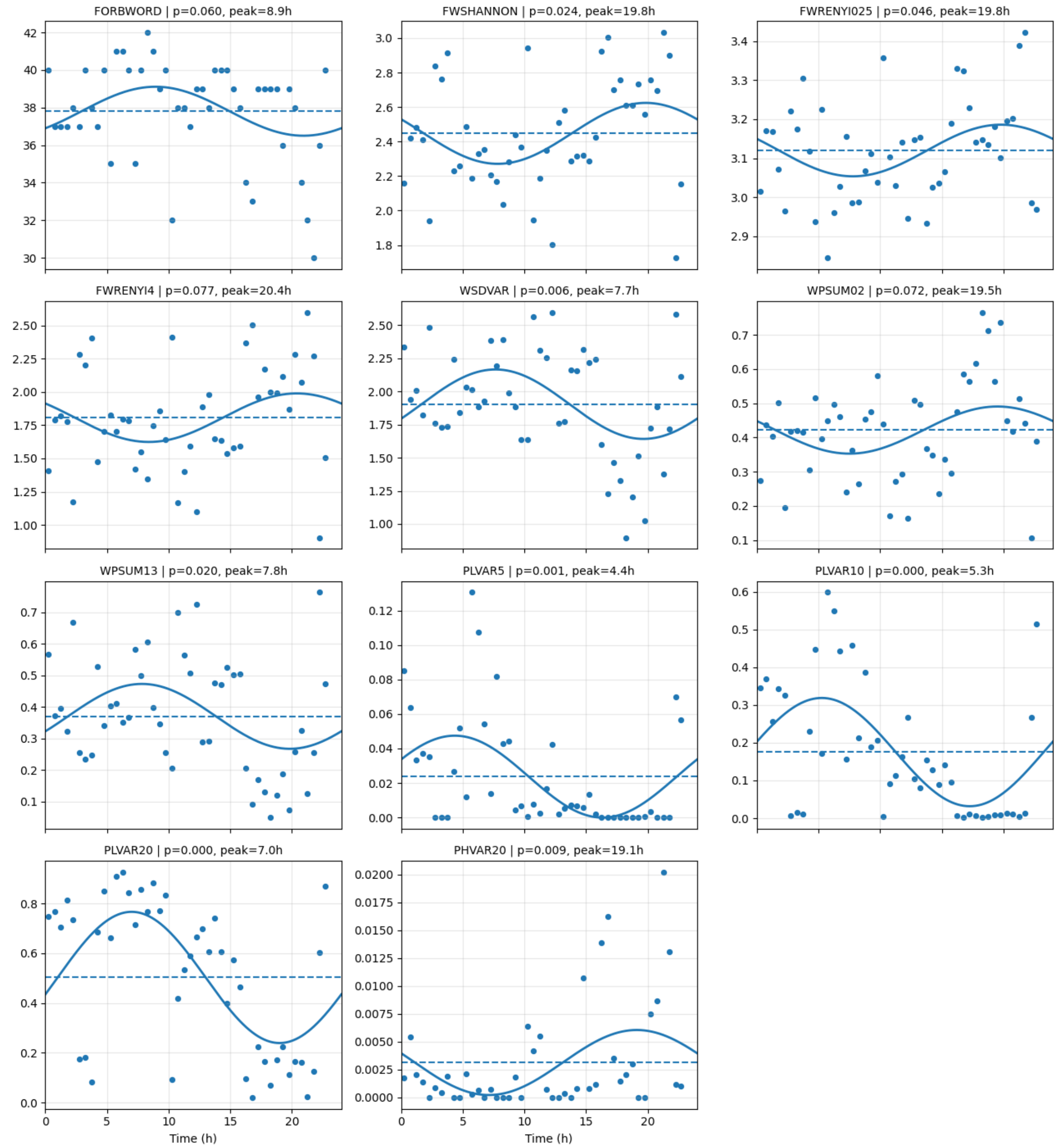

Figure S4. Example of Cosinor modeling on symbolic dynamics indices. The Cosinor model was primarily used to provide a compact, standardized description of circadian variation in symbolic dynamics indices, facilitating comparisons with prior chronobiological studies. These indices typically exhibit smoother and more regular 24-hour oscillatory patterns, making them more amenable to first-harmonic Cosinor approximation. In contrast, entropy-based measures often show more complex, non-sinusoidal, and potentially multimodal temporal dynamics that are not fully captured by a single-component Cosinor model. For this reason, we opted not to apply Cosinor analysis equally across all metrics.
