# Supplementary material for: Temporal Properties of Cardiorespiratory Coupling in Patients with Heart Failure During the Circadian Cycle
Source: Entropy (Basel). 2026 May 6;28(5):524. doi: 10.3390/e28050524 (PMC13205341; doi:10.3390/e28050524)
Supplement: Supplementary file 1 [file entropy-28-00524-s001.zip › entropy-4150519-supplementary.pdf]

# Supplementary tables

**Table S1.** Multivariate regression analysis of heart rate variability and entropy-related metrics: effects of group, age, sex, and beta-blocker treatment

| Variable | Beta_Group | p_Group | Beta_Age | p_Age | Beta_Bblock | p_Bblock | N | R2 | p_Group_FDR | p<0.05 | FDR<0.05 |
|---|---|---|---|---|---|---|---|---|---|---|---|
| **meanNN** | 96.54588449 | 0.00624209 | 1.397653 | 0.313043 | -10.62859 | 0.794488 | 163 | 0.105805 | 0.025860072 | TRUE | TRUE |
| **sdNN** | -1.24367861 | 0.8897957 | -0.66358 | 0.063685 | -28.505187 | 0.00727 | 163 | 0.199707 | 0.889795701 | FALSE | FALSE |
| **cvNN** | -0.01479796 | 0.15790567 | -0.00104 | 0.012871 | -0.0302089 | 0.014326 | 163 | 0.238494 | 0.241013911 | FALSE | FALSE |
| **sdaNN1** | -8.51472062 | 0.30043831 | -0.60547 | 0.064311 | -23.981914 | 0.01338 | 163 | 0.236735 | 0.348508443 | FALSE | FALSE |
| **sdaNN5** | -10.9284186 | 0.17962312 | -0.598 | 0.064733 | -19.982534 | 0.036683 | 163 | 0.229515 | 0.248050972 | FALSE | FALSE |
| **sdaNN10** | -11.0172609 | 0.17672137 | -0.58337 | 0.071873 | -19.684044 | 0.039811 | 163 | 0.224688 | 0.248050972 | FALSE | FALSE |
| **rmssd** | 6.670229848 | 0.01447452 | -0.42511 | 0.000107 | -3.8974733 | 0.218556 | 163 | 0.16857 | 0.05196757 | TRUE | FALSE |
| **pNN50** | 0.046289926 | 0.00494728 | -0.00283 | 2.01E-05 | -0.0225242 | 0.237409 | 163 | 0.181513 | 0.025513633 | TRUE | TRUE |
| **pNN100** | 0.014729283 | 0.00321352 | -0.00073 | 0.000253 | -0.0087462 | 0.130691 | 163 | 0.258572 | 0.025513633 | TRUE | TRUE |
| **pNN200** | 0.002776741 | 0.00527868 | -6.1E-05 | 0.118748 | -0.0023847 | 0.039399 | 163 | 0.173229 | 0.025513633 | TRUE | TRUE |
| **shannon** | -0.09562049 | 0.22101114 | -0.00508 | 0.10165 | -0.3089336 | 0.000872 | 163 | 0.227724 | 0.291332868 | FALSE | FALSE |
| **renyi025** | -0.02240878 | 0.75361788 | -0.00589 | 0.038873 | -0.2205861 | 0.008967 | 163 | 0.168057 | 0.780532805 | FALSE | FALSE |
| **renyi4** | -0.13156949 | 0.11732859 | -0.00464 | 0.163566 | -0.3461922 | 0.000522 | 163 | 0.24116 | 0.212658071 | FALSE | FALSE |
| **renyi2** | -0.12020188 | 0.14456385 | -0.00472 | 0.148437 | -0.3434119 | 0.000455 | 163 | 0.241245 | 0.241013911 | FALSE | FALSE |
| **pNNl10** | -0.04966679 | 0.1530033 | 0.003818 | 0.006027 | 0.04825996 | 0.234687 | 163 | 0.077821 | 0.241013911 | FALSE | FALSE |
| **pNNl20** | -0.05749788 | 0.09077243 | 0.004853 | 0.000394 | 0.0363211 | 0.359438 | 163 | 0.111304 | 0.175493359 | FALSE | FALSE |
| **pNNl30** | -0.06175534 | 0.0260501 | 0.004475 | 6.46E-05 | 0.033037 | 0.305698 | 163 | 0.140792 | 0.058111755 | TRUE | FALSE |
| **pNNl50** | -0.04639166 | 0.00526993 | 0.00288 | 1.76E-05 | 0.02256025 | 0.241179 | 163 | 0.18203 | 0.025513633 | TRUE | TRUE |
| **ULF** | 0.69293597 | 0.63894315 | -0.01516 | 0.795678 | -0.1308102 | 0.939616 | 163 | 0.007294 | 0.712667355 | FALSE | FALSE |
| **VLF** | 1.109265402 | 0.09036776 | 0.004297 | 0.868007 | -1.8590952 | 0.01573 | 163 | 0.040829 | 0.175493359 | FALSE | FALSE |
| **LF** | 0.031636492 | 0.75200875 | -0.0118 | 0.003356 | -0.2565784 | 0.029634 | 163 | 0.137581 | 0.780532805 | FALSE | FALSE |
| **HF** | 0.133157899 | 0.02010593 | -0.00165 | 0.464334 | -0.1487803 | 0.026275 | 163 | 0.045386 | 0.053006554 | TRUE | FALSE |
| **P** | 1.966993653 | 0.24945895 | -0.02432 | 0.719037 | -2.3952639 | 0.230591 | 163 | 0.018967 | 0.314535198 | FALSE | FALSE |
| **UVLF** | 1.802201349 | 0.27666543 | -0.01086 | 0.868405 | -1.9899053 | 0.304338 | 163 | 0.015909 | 0.334304063 | FALSE | FALSE |
| **LFn** | -0.09944664 | 0.00024989 | -0.00075 | 0.475231 | -0.062873 | 0.044451 | 163 | 0.244554 | 0.003623467 | TRUE | TRUE |
| **HFn** | 0.099446638 | 0.00024989 | 0.000753 | 0.475232 | 0.06287298 | 0.044451 | 163 | 0.244554 | 0.003623467 | TRUE | TRUE |

| Variable | Parameter | Beta_Group | p_Group | Beta_Age | p_Age | Beta_Sex | p_Sex |
|---|---|---|---|---|---|---|---|
| **FORBWORD** | Mesor | -0.44241 | 0.692345 | 0.2712 | 1.7E-14 | -1.83706 | 0.125164 |
| **FORBWORD** | Amplitude | 0.718569 | 0.276839 | -0.05903 | 0.029046 | 0.32619 | 0.290168 |
| **FWSHANNON** | Mesor | -0.05202 | 0.243077 | -0.00737 | 4.29E-05 | 0.106721 | 0.01163 |
| **FWSHANNON** | Amplitude | 0.015521 | 0.511459 | -0.00325 | 0.000469 | 0.028271 | 0.008346 |
| **FWRENYI025** | Mesor | -0.05864 | 0.235909 | -0.00774 | 5.66E-05 | 0.05316 | 0.137652 |
| **FWRENYI025** | Amplitude | 0.030843 | 0.020192 | -0.00111 | 0.000105 | 0.009199 | 0.555506 |
| **FWRENYI4** | Mesor | -0.04262 | 0.172606 | -0.00519 | 0.003088 | 0.098864 | 0.012773 |
| **FWRENYI4** | Amplitude | 0.001981 | 0.942118 | -0.00429 | 0.00032 | 0.037127 | 0.027457 |
| **WSDVAR** | Mesor | -0.26127 | 6.79E-06 | -0.00917 | 2.98E-05 | -0.00958 | 0.831867 |

| | | | | | | | |
|---|---|---|---|---|---|---|---|
| **WSDVAR** | Amplitude | -0.0184 | 0.454564 | 0.00154 | 0.029953 | 0.041906 | 0.045581 |
| **WPSUM02** | Mesor | 0.077642 | 0.001188 | 0.005821 | 6.6E-08 | -0.01719 | 0.360414 |
| **WPSUM02** | Amplitude | 0.003114 | 0.73682 | 0.001023 | 0.014249 | 0.014002 | 0.101906 |
| **WPSUM13** | Mesor | -0.07055 | 6.44E-05 | -0.00248 | 0.002008 | -0.01651 | 0.258192 |
| **WPSUM13** | Amplitude | -0.01694 | 0.059203 | -0.00044 | 0.255307 | 0.017791 | 0.02489 |
| **PLVAR5** | Mesor | 0.015509 | 0.057383 | -0.00023 | 0.416841 | 0.004513 | 0.23699 |
| **PLVAR5** | Amplitude | 0.009679 | 0.032092 | -0.00012 | 0.257928 | 0.000818 | 0.84895 |
| **PLVAR10** | Mesor | 0.022419 | 0.293962 | 0.000695 | 0.455131 | -0.00717 | 0.710332 |
| **PLVAR10** | Amplitude | 0.00485 | 0.548368 | 5.44E-05 | 0.805985 | -0.00028 | 0.975594 |
| **PLVAR20** | Mesor | -0.00617 | 0.859235 | 0.00445 | 0.000182 | -0.05138 | 0.159761 |
| **PLVAR20** | Amplitude | -0.01901 | 0.091973 | 2.35E-05 | 0.973267 | 0.001099 | 0.938319 |
| **PHVAR20** | Mesor | 0.020451 | 5.26E-05 | -0.00139 | 1.38E-10 | 0.001456 | 0.815476 |
| **PHVAR20** | Amplitude | 0.004598 | 0.28846 | -0.00109 | 6.55E-10 | -0.0034 | 0.392032 |
| **PHVAR50** | Mesor | 0.00359 | 0.001808 | -0.00019 | 3.32E-08 | -0.00045 | 0.67115 |
| **PHVAR50** | Amplitude | 0.002343 | 0.064763 | -0.0002 | 0.000183 | -0.00152 | 0.134883 |

**Table S2.** Acrophase concentration test

| Variable | N_control | N_HF | R_control | R_HF | R_diff(HF-control) | Boot_mean_diff | CI2.5 | CI97.5 | Significant |
|---|---|---|---|---|---|---|---|---|---|
| FORBWORD | 88 | 75 | 0.426239 | 0.090996 | -0.33524 | -0.30123 | -0.48842 | -0.09643 | TRUE |
| FWRENYI025 | 88 | 75 | 0.365546 | 0.089784 | -0.27576 | -0.24119 | -0.42658 | -0.03226 | TRUE |
| FWRENYI4 | 88 | 75 | 0.619216 | 0.207248 | -0.41197 | -0.40149 | -0.59688 | -0.20455 | TRUE |
| FWSHANNON | 88 | 75 | 0.588806 | 0.141495 | -0.44731 | -0.42524 | -0.60512 | -0.22729 | TRUE |
| PHVAR20 | 88 | 75 | 0.589394 | 0.357162 | -0.23223 | -0.22893 | -0.43235 | -0.01931 | TRUE |
| PHVAR50 | 88 | 75 | 0.626283 | 0.473109 | -0.15317 | -0.15417 | -0.33045 | 0.023162 | FALSE |
| PLVAR10 | 88 | 75 | 0.584835 | 0.315128 | -0.26971 | -0.26669 | -0.4521 | -0.08394 | TRUE |
| PLVAR20 | 88 | 75 | 0.606943 | 0.282714 | -0.32423 | -0.31922 | -0.51436 | -0.12424 | TRUE |
| PLVAR5 | 88 | 75 | 0.513519 | 0.238805 | -0.27471 | -0.26772 | -0.47225 | -0.065 | TRUE |
| WPSUM02 | 88 | 75 | 0.586249 | 0.237097 | -0.34915 | -0.34083 | -0.52591 | -0.15915 | TRUE |
| WPSUM13 | 88 | 75 | 0.835523 | 0.370164 | -0.46536 | -0.46039 | -0.62338 | -0.29885 | TRUE |
| WSDVAR | 88 | 75 | 0.81756 | 0.357283 | -0.46028 | -0.45431 | -0.61461 | -0.29566 | TRUE |

**Table S3.** Subject-level Spearman correlation between coordination percentage and $FuzzEn_{PRQ}$

Correlation between subject-averaged coordination percentage and subject-averaged $FuzzEn_{PRQ}$ across time windows (24 h, day, night) and ε tolerances. Values are Spearman's ρ with bootstrap 95% CI.

**(A) All subjects (HF + Control)**

| Window | ε (s) | ρ | 95% CI | p |
|---|---|---|---|---|
| 24 h | 0.2 | 0.083 | [-0.073, 0.240] | 0.2855 |
| 24 h | 0.1 | 0.022 | [-0.136, 0.175] | 0.7801 |
| Day | 0.2 | 0.172 | [0.020, 0.325] | **0.0252** |
| Day | 0.1 | 0.141 | [-0.020, 0.294] | 0.0668 |
| Night | 0.2 | -0.002 | [-0.150, 0.148] | 0.9753 |
| Night | 0.1 | -0.069 | [-0.224, 0.093] | 0.3715 |

**(B) Stratified by group**

| Group | Window | ε (s) | ρ | 95% CI | p |
|---|---|---|---|---|---|
| HF | 24 h | 0.2 | 0.146 | [-0.089, 0.373] | 0.1906 |
| HF | 24 h | 0.1 | 0.064 | [-0.177, 0.293] | 0.5683 |
| HF | Day | 0.2 | 0.275 | [0.044, 0.487] | **0.0125** |
| HF | Day | 0.1 | 0.226 | [-0.014, 0.457] | **0.0412** |
| HF | Night | 0.2 | -0.039 | [-0.272, 0.194] | 0.7312 |
| HF | Night | 0.1 | -0.131 | [-0.353, 0.107] | 0.2425 |
| Control | 24 h | 0.2 | -0.131 | [-0.320, 0.071] | 0.2276 |
| Control | 24 h | 0.1 | -0.120 | [-0.313, 0.079] | 0.2686 |
| Control | Day | 0.2 | -0.119 | [-0.319, 0.079] | 0.2736 |
| Control | Day | 0.1 | -0.092 | [-0.282, 0.109] | 0.3938 |
| Control | Night | 0.2 | 0.056 | [-0.155, 0.255] | 0.6040 |
| Control | Night | 0.1 | 0.041 | [-0.165, 0.243] | 0.7081 |
